# A Review of Multiple Scale Fibrous and Composite Systems for Heating Applications

**DOI:** 10.3390/molecules26123686

**Published:** 2021-06-16

**Authors:** Inês Pimentel Moreira, Usha Kiran Sanivada, João Bessa, Fernando Cunha, Raul Fangueiro

**Affiliations:** 1 Centre for Textile Science and Technology (2C2T), University of Minho, 4800-058 Guimarães, Portugal; joaobessa@fibrenamics.com (J.B.); fernandocunha@det.uminho.pt (F.C.); 2 Department of Mechanical Engineering, University of Minho, 4800-058 Guimarães, Portugal; ushakiran.sanivada@gmail.com

**Keywords:** heating, Joule effect, polymers, fibers, composites, textiles, thermal conductivity

## Abstract

Different types of heating systems have been developed lately, representing a growing interest in both the academic and industrial sectors. Based on the Joule effect, fibrous structures can produce heat once an electrical current is passed, whereby different approaches have been followed. For that purpose, materials with electrical and thermal conductivity have been explored, such as carbon-based nanomaterials, metallic nanostructures, intrinsically conducting polymers, fibers or hybrids. We review the usage of these emerging nanomaterials at the nanoscale and processed up to the macroscale to create heaters. In addition to fibrous systems, the creation of composite systems for electrical and thermal conductivity enhancement has also been highly studied. Different techniques can be used to create thin film heaters or heating textiles, as opposed to the conventional textile technologies. The combination of nanoscale and microscale materials gives the best heating performances, and some applications have already been proven, even though some effort is still needed to reach the industry level.

## 1. Introduction

**Figure 1:**
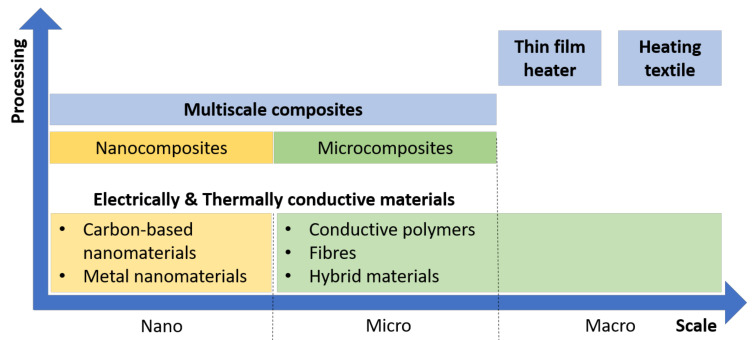
Schematic diagram of the multiscale approaches to heating technologies.

Heating technologies have been highly studied lately for distinct applications that go from smart textiles/wearables and thermotherapy to defreezing/defogging applications. Heating is considered passive when it depends on light irradiation of solar energy. As previously reviewed [1], photothermal heating uses inorganic nanomaterials, semiconducting polymers or ceramics to emit absorbed energy as heat. In contrast, active heaters can be developed by using conductive materials, relying on the Joule heating upon the application of low voltages. They can be produced in different configurations that go from thin film heaters to heating textiles, and from nano- to macroscale (Figure 1).

All the technologies end up using the same kind of conductive materials. Apart from metals and metal oxides, metallic nanostructures, carbon-based materials and inherently conductive polymers are highly promising and have been used in several research studies. Different techniques have been explored throughout the years to improve the thermal and/or electrical conductivity of materials, including the addition of fillers to a polymeric matrix. These fillers can be in the form of fibers, particles or flakes, homogeneously distributed within the polymer matrix [2].

This review is organized as follows: Section 2 will focus on the scientific principles behind the development of active heaters via the Joule effect; Section 3 is on the emerging nano and micromaterials that present electrical and/or thermal conductivity; Section 4 will be about fibrous systems; Section 5 focuses on the combination of the intrinsically conductive materials from Section 3 into composites of different scales to improve thermal conductivity, electrical conductivity or both; Section 6 is on the development of thin film heaters; Section 7 is focused on heating textiles; Section 8 will address the applications of these heating systems; and finally, Section 9 will conclude and refer future prospects.

## 2. Scientific Principles

### 2.1. Joule Effect—Electrical Conductivity

Joule heating involves energy dissipation in the form of heat upon the passage of an electric current. For heating technologies based on the Joule effect, conductive materials must be used, which conduct electricity while offering resistance to the flow. Joule’s law states that the generated heat Q depends on the electric current I that passes through a homogeneous conductive material with resistance R per unit of time t:

(1) $$Q=I^{2}\mathrm{Rt}\Leftrightarrow P=I^{2}R$$

So the total power consumption P of a heater can be calculated by these electrical formulas. The electrical resistance R is the measure of opposition to the electric current and depends on the resistivity ρ, inherent characteristic of the material, and on the length L and area A.

(2) $$R=\;\rho \frac{L}{A}$$

When replacing, this gives:

(3) $$Q=I^{2}\rho \frac{L}{A}t$$

Electrical conductivity σ is the inverse of electrical resistivity and is thus a key aspect for a Joule heating system to be created.

### 2.2. Heat Transfer—Thermal Conductivity

Heat transfer can occur through three physical phenomena: convection, radiation and conduction. Convection comprises heat transfer from one place to another through the movement of fluids. It can be natural due to the density gradient of a fluid caused by the temperature difference, or it can be forced and generated by external pumps, fans or air conditioners. However, air convection is the main path of heat dissipation, increasing with temperature. A steady state temperature is reached when Joule heating and convection reach a dynamic balance at the elevated temperature. In turn, radiation relies on energy transfer in the form of photons in electromagnetic waves, due to the thermal motion of charged particles in matter. Thermal conduction depends on the transfer of vibrational energy from one particle to the adjacent one in a material. The ability of a material to conduct heat is given by the thermal conductivity (κ) and is described by Fourier’s Law. This states that when a temperature gradient exists within a body, heat energy will flow from the higher to the lower temperature. The thermal conductivity can be measured by different methods, with the SI units given in Watts per meter Kelvin (Wm^−1^K^−1^). These measurement methods can be the guarded hot plate, unguarded hot plate, heat flow meter, cylindrical probe method, hot wire method, laser flash method, step method, transient slip, and transient plane method [3]. The most commonly used one is the laser flash diffusivity method [2], as it is easier to measure the diffusivity than the thermal conductivity. In this, the temperature rise of a sample’s rear face is recorded throughout the time when the front surface of a heated plane-parallel sample is heated by a light pulse. A material with a higher thermal conductivity conducts heat better than a material with a larger thermal resistivity.

**Figure 2:**
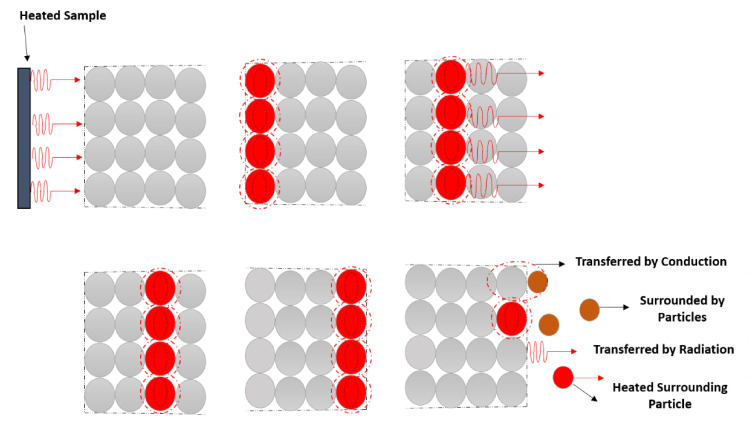
Schematic representation of thermal conductance in a crystalline material. Adapted from [6].

In solids, phonons, electrons or photons are the three types of carriers responsible for this energy transfer from a higher to a lower temperature area [4]. However, the mechanisms by which thermal conduction occurs vary from metals to non-metals. Free electrons are the main carriers acting within metals, which explains why metals with high electrical conductivity also present high thermal conductivity. On the other side, the thermal conduction within non-metals occurs mainly by phonons, which are artificially quantized solid lattice vibrations [5]. In crystalline materials, the most thermally conductive materials, the vibration within the rigid crystal lattice normally causes phonon transfer. When the heat source encounters one side of the crystal lattice, the heat is conducted through the atoms in the first layer in the form of vibrations [4,6]. Since the atoms are closely packed and there are strong bonds between them, the vibrations are passed quickly through the atoms. These are the reasons why heat conductance is so rapid in materials with a densely packed lattice. The heat transfer mechanism in crystalline materials is pictorially represented in Figure 2.

**Figure 3:**
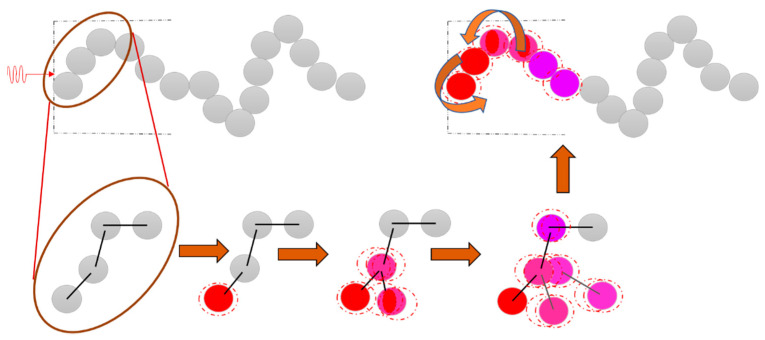
Thermal conductance mechanism in a polymer. Adapted from [6].

In most polymers, phonons are also the main carriers as there is no free electron movement [7]. However, it follows a complex process and factors such as crystallinity, temperature, orientation of macromolecules, among others, influence the thermal conduction in a polymer [4]. Transfer of heat will take place when the polymer’s surface meets the heating source. The heat transfers from the first atom to the last atom in the polymer molecular chains through vibration and it does not propagate in the form of a wave. The possibility of induced disordered vibration and rotation of atoms when the heat is transferred in the molecular chain can result in a significant reduction in the thermal conductivity of a polymer [4,6]. The thermal conductance phenomenon within a polymer is illustrated in Figure 3.

The thermal conductivity of polymers, in general, is observed to be around 0.2 Wm^−1^K^−1^ (see Table 1), and hence it becomes necessary to improve it for polymers to be used. In fact, there is a quest for improving their thermal conductivity for various applications. The process of thermal conduction within crystalline materials, but also within composites, has been carefully reviewed before [6,8]. A good heat conductor should have a lattice structure where atoms are very close to each other to allow for a quick heat transfer from the first into the last atom.

In order to create the Joule effect, heating systems must present a high electrical conductivity, while a high thermal conductivity is also critical to ensure the generated heat is homogeneous.

## 3. Intrinsically Conductive Materials

Different raw materials with high electrical and/or thermal conductivity can be used for heating technologies. We hereby present them and their main properties, from nano- to macroscale: carbon-based and metallic-based nanomaterials and intrinsically conductive polymers, among others. They can be used independently or as hybrids. The use of fibrous systems and composite systems, by the combination of these conductive materials with polymers, is later reviewed in Section 4 and Section 5.

### 3.1. Carbon-Based Nanomaterials

Despite being relatively recent, there are numerous studies on the use of carbon-based nanomaterials for practical applications due to their electrical, optical and thermal properties. In particular, graphene and carbon nanotubes have been used for thermal engineering thanks to their high thermal conductivity (see Table 2) [12]. These are two carbon allotropes with sp^2^-hybridized carbon atoms but different structures. Du et al. published a detailed review that explains the fundamentals of graphene and carbon nanotubes (CNTs) [13], in addition to their application on transparent conductive films for optoelectronic devices.

The newly discovered graphene is a 2-dimensional sheet of carbon atoms arranged in a honeycomb lattice. Between the carbon allotropes, graphene presents the highest thermal conductivity (around 5000 W m^−1^ K^−1^ for single-layer graphene at room temperature). Due to its unique chemical structure, with a densely packed carbon atom arrangement, graphene thermal conduction is thought to occur through phonon waves [4]. This explains why its thermal conductivity is one of the highest of all materials (Table 2), in addition to the fact that it also presents an ultrahigh specific surface area. Graphene nanoplatelets (GNPs) are short stacks of graphene sheets with a platelet shape. Depending on the fabrication method, GNPs can vary in size and thickness and thus the thermal and electrical conductivities can vary considerably. In turn, stacked graphene forms the 3D graphite, which is abundantly available in nature. The atoms are bonded by covalent bonds, the distance between the bonds is approximately 0.412 nm and the distance between the layers of approximately 0.335 nm [23]. Its sp^2^ configuration explains its high in-plane thermal conductivity [9]. It can be modified into various forms, namely expanded graphite (EG), graphite nanoplatelets, and graphite flakes (GF), that are easily dispersed in polymers [23]. EG is a light and worm-like structure with a thickness of 100 to 400 nm, formed when the graphite-intercalated compounds are exposed to a sudden temperature increase for a short time. Exfoliation of EGs through ultrasonication will produce graphite nanoplatelets, which consist of stacks of graphite layers. The thickness of the graphite nanoplatelets is less than 10 nm and the diameter is in the range of sub-micron to approximately 15 µm [24].

Single-walled carbon nanotubes (SWCNT) have a cylindrical nanostructure formed by rolled-up graphene. They arose as promising for thermal management applications, with thermal conductivity values depending on many factors. Pop and co-workers developed a theoretical study on an individual SWCNT, reporting a thermal conductivity of nearly 3500 Wm^−1^K^−1^ at room temperature [25].

### 3.2. Metallic-Based Nanomaterials

Metals such as aluminum, copper, gold, silver and others, appear as the obvious materials for heating due to their high electrical and thermal conductivities (Table 2). Among all metals, silver presents the highest electrical (6.3 × 10^7^ Sm^−1^) and thermal conductivity (427 W m^−1^ K^−1^) at room temperature. Copper follows silver in terms of properties, but it is less stable in air. In turn, the cost of silver is lower than that of gold and platinum. The engineering of metal nanostructures has attracted a lot of research lately, as a way to manipulate photons and electrons and to produce transparent electrodes for optoelectronic devices, as will be explored later in this review. The main metallic-based nanostructures are metallic meshes, metallic grids and metallic nanowires. Even though the difference between metallic meshes and grids is sometimes confusing in the literature, we hereby consider that metallic meshes do not present an organized conductive direction, while metallic grids present a periodic arrangement of lines.

Metal nanoparticles have unique properties, mainly due to their dimensions and subsequent large ratio of atoms in the particle surface. As opposed to metal nanoparticles, metal nanofibers or metal nanowires present elongated structures that help the electrical current passage. That is the reason why they have been largely investigated as network electrodes for optoelectronic devices, with strictly controlled electrical and optical properties. Copper nanofibers provide around 2–3 orders of magnitude higher aspect ratios than other 1D nanomaterials such as carbon nanotubes or silver nanowires. Silver nanowires have been the most explored nanomaterials, with copper nanowires appearing as an alternative. Some determinant aspects as their dispersing agents, wire geometry and aspect ratio have been fully studied and reviewed over the years [26]. The junctions between the wires in the network are crucial for the conductivity paths, with a well-sintered junction causing a lower junction’s electrical resistance. This leads to overall lower electrical resistance and enhanced electrical and mechanical properties. The thermal conductivity of silver nanowires at room temperature was reduced by 55% from that of bulk silver (see Table 2). Cheng et al. used a unified thermal resistivity method to elucidate the electron scattering mechanism of bulk and silver nanostructures [18]. In bulk silver, the phonon scattering dominates the electron transport, with rare structural scatterings, while in metallic nanostructures, there are grain boundary and surface scatterings. These scatterings limit the electron free path, which contributes to the reduction in thermal and electrical conductivity.

### 3.3. Intrinsically Conductive Polymers

A broad range of available polymers has been documented to have significant electrical conductivity with the addition of dopants. This included polyacetylene, polypyrrole, polyaniline and polythiophene, among others. After polyacetylene has been disregarded commercially due to instability in air, polyaniline and polythiophene have emerged as the most promising in both research and industry, not only due to their stability but also due to their dissolution ability in common solvents. Conductive polymers have attracted much attention due to their advantages, such as recyclability, light weight, low cost, chemical stability and easy processability [19]. Their solution processability, in particular, makes intrinsically conductive polymers highly attractive for flexible electronic devices. The fact that the polymer’s molecular structure can be tuned to control the electrical and mechanical properties of the resulting material is also highly promising.

The correlation between electrical and thermal conductivities in conductive polymers largely differs from the ones in metals, since electrons are not the carriers for electrical nor thermal current [19]. In contrast to the electrically insulating polymers, the thermal conductivity in conductive polymers happens through phonons and charge carriers. As for the insulating polymers, the thermal conductivity in a conductive polymer strongly depends on numerous factors, such as defects or structural faults, its chemical components, side group molecular weight, molecular density distribution, temperature, processing conditions, and others [7]. The presence of defects in polymers can cause phonon scattering at the interface between amorphous and crystalline states, leading to low thermal conductivities [4]. The low thermal conductivities (Table 2) of electrically conductive polymers are thus explained by the same reasons pointed to before. There are a few intrinsically conductive polymers, with a balance between crystallinity and low insulating content, that can be used as conductive thin films or as conductive fillers in nanocomposites [27]. However, engineering of the thermal conductivity is not an easy task, as for crystalline materials, as it depends on several structural factors such as the type and size of fillers, conformation of polymer chains and chain structure [19].

Polythiophene materials, namely Poly(3,4-ethylenedioxythiophene (PEDOT) doped with aqueous polystyrenesulfonate (PSS) (PEDOT:PSS), appeared as one of the most promising intrinsically conductive polymers. It presents the highest reported conductivity among solution-processed polymers [28], which has been reported to increase from 0.8 Scm^−1^ to 80 Scm^−1^ in the presence of the solvent DMSO [29]. Indeed, some studies have been published on different techniques to enhance the electrical conductivity of PEDOT:PSS [29,30], including organic solvent, surfactant, salt solution treatments, and others. Romasanta and colleagues have reported a microfluidic method of adding a secondary liquid dopant to alter PEDOT:PSS films locally, boosting the electrical conductivity in a specific area to take advantage of the localized Joule’s effect [31].

### 3.4. Hybrid Nanomaterials

Hybrid nanomaterials synergistically combine materials of different natures, such as organic and inorganic. They can be highly promising structures that keep the beneficial properties of both inorganic nanoparticles and polymers, e.g., [32]. In addition, multifunctional hybrid nanomaterials can be produced, with the ability to tune their properties through the combination of functional components [33].

## 4. Fibrous Systems

Conductive fibers have been largely exploited to conduct electricity and provide heating via the Joule effect, but also for sensing and for providing antimicrobial and electromagnetic shielding (EMS) properties.

Carbon fibers have a diameter of 5–10 µm and are composed of carbon atoms bonded together to form a long chain. They have been used in applications in the aerospace and automotive industries, among others, due to their high stiffness, tensile strength, chemical resistance and temperature tolerance. In fact, they present a high ratio of strength to weight and a high ratio of modulus to weight. In addition to their large thermal conductivity (Table 2), they also present a large electrical conductivity (>3300 S/cm) [14]. Even though they are under the category of carbon-based materials in Table 2, they are micromaterials. In addition to providing strength and stiffness to composites, these carbon fibers also introduce new functionalities such as thermal and electrical conductivity, based on their intrinsic properties. The most common types of carbon fibers are polyacrylonitrile (PAN) and pitch carbon fiber [34]. The pitch-based CFs exhibit a thermal conductivity of about 1000 Wm^−1^K^−1^ [35], much higher than that of PAN (8–12 W m^−1^ K^−1^) [36].

Metallic fibers are manufactured fibers composed of metals, either plastic-coated metal, metal-coated plastic or a core completely covered by metal. Whole metallic filaments are fabricated by a continuous heat treatment applied to the metallic wires followed by drawing [37]. Since 1968, Underwood’s technique of producing metallic yarns by spinning a bundle of metallic fibers has been followed [38]. For that, a solid mass of metal is shredded to produce elongated filaments with rough or serrated surfaces. Stainless steel fibers can be used to produce fine stainless-steel yarns, suitable for knitting or weaving and largely used in different heating applications.

The morphological properties of fibrous systems can highly influence the thermal and electrical transport performance. It has been reported that fiber length, diameter and orientation of natural fibers affect mechanical, thermal properties, among others [39].

## 5. Composite Systems

Composites have been largely exploited and studied for improved mechanical properties, having received special attention in recent years [40,41,42] for these reasons. However, this section focuses on the development of composites for improved thermal or/and electrical conductivities. The composites can be categorized as nanocomposites, microcomposites and multiscale composites depending on the used fillers, among the previously introduced intrinsically conductive materials.

### 5.1. Composites for Improved Thermal Conductivity

As mentioned above, the low thermal conductivity of standard polymers (Table 1) hinders their usage in thermal management applications. For this purpose, the creation of composites by incorporating different types of fillers has been consistently used in the literature. A short review has recently focused on the rational design of conductive fillers for the production of thermally conductive polymer composites [43]. The thermal conductivity of the fillers has been discussed before (Table 2) and depends on the way the heat transfers: when it happens through photons, the fillers have a low thermal conductivity value; if the transfer of heat is due to electrons, the fillers present a high thermal conductivity value. We will review the different approaches used in the literature regarding the improvement of thermal conductivities.

#### 5.1.1. Nanocomposites for Improved Thermal Conductivity

Through the addition of a nanofiller, which is intrinsically thermally conductive, the production of polymers with combined properties is made possible. As mentioned before, the free electrons transfer the heat and move at high speeds, explaining the good thermal conductivity of metallic and carbon-based materials [3]. Carbon-based fillers, such as carbon nanotubes (CNTs), graphite and graphene nanoplatelets (GNPs), are the most used nanofillers, owing to their good thermal conductivities (Table 2). These nanofillers are incorporated into polymers to produce highly conductive polymer matrix composites (PMCs) [44]. As per the literature, there are three ways to produce a composite with good thermal conductivity. The first method includes the dispersion of powders or nanoparticles with high thermal conductivity into low thermal conductivity base material. The second method includes a combination of porous material with high conductivity with a base material. In turn, the third method includes the high thermally conductive stationary structure with extended surface embedded into the base material [45]. Different theories describe the thermal conductive mechanisms of thermally conductive polymer composites, such as the thermal conductive path, the thermal conductive percolation and the thermoelastic coefficient theory. The most popular theory among them is the thermal conductive path theory, where the addition of fillers to an extent will form a thermal conductive network that facilitates thermal conductivity improvement [5]. Heat transfer paths are formed when the fillers are added beyond a certain volume fraction, and this phenomenon is known as percolation, whereby a sudden increment in the composite thermal conductivity is observed [46]. The thermoelastic coefficient theory is based on the heat conduction due to the phonons, with the thermal conductivity of the composites depending on the efficiency of phonon transfer. These phonons influence the materials’ properties such as specific heat capacity, thermal expansion coefficient and thermal conductivity [47].

**Figure 4:**
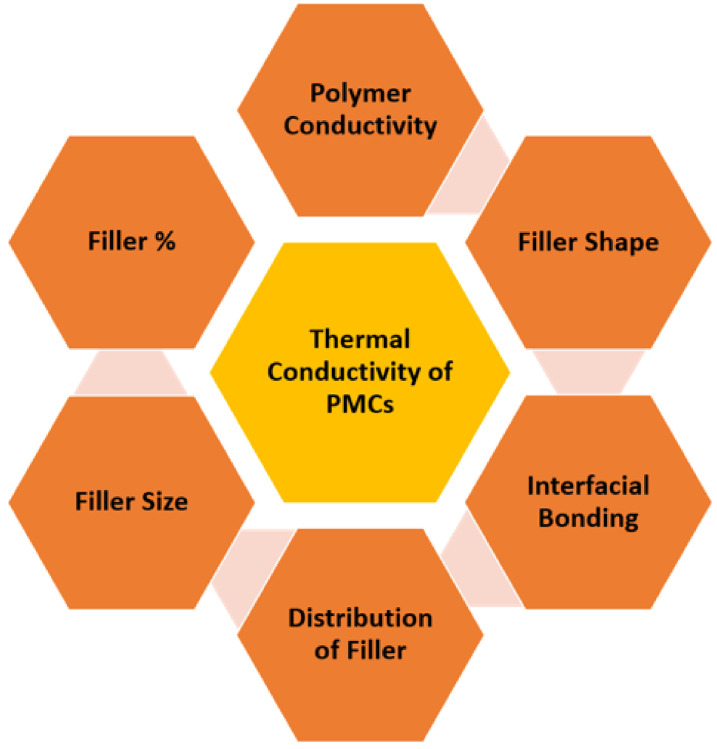
Schematic representation of factors that influence the thermal conductivity of PMCs.

The thermal conductivity of the formed PMC depends on a number of factors, schematically represented in Figure 4: intrinsic polymer conductivity, filler conductivity, volume fraction, filler shape and size, distribution, interfacial bonding between filler and matrix. When using composites that contain nanofillers with a high aspect ratio, the percolation threshold is low. That explains why the composites with graphene or nanotubes possess higher thermal conductivity at low volume fractions [48]. The shape of the fillers also plays a crucial role, whereby the fillers with platelet shape are more effective in comparison to spherical and cylindrical fillers. This could be attributed to the large contact area, which facilitates the contact between the fillers adjacent to one another [3,16]. Since a large interface thermal resistance results in a lower thermal conductivity, it must be minimized in order to improve thermal conductivities [49]. As previously mentioned, the molecular structure, crystallinity, and orientation of polymer chains are also important parameters that influence the thermal conductivity of a polymer [3]. Improving the thermal conductivity by engineering the interchain interactions was reported in a study where the blending of two polymers created a homogeneously distributed thermal network, having achieved a thermal conductivity value of 1.5 W m^−1^ K^−1^ [50].

- Addition of one nanofiller

Several researchers have successfully improved polymers’ thermal conductivity by the addition of nanofillers such as carbon nanotubes, graphene oxide, reduced graphene oxide, graphene nanoplatelets, etc. The improvement was due to the formation of efficient networks that conduct the thermal energy. It was observed from the literature that the thermal conductivity is enhanced with an increased loading of nanofillers, even though it is challenging to achieve higher loadings without increasing the viscosity of the polymer. Some works reported different methodologies to improve the thermal conductivity, as reviewed in this paper. The use of graphene-filled polymer composites for the enhancement of thermal conductivity has been carefully reviewed [4,51]. Among the fillers, GNPs were observed to achieve the best values of thermal conductivity, which is due to their 2D shape and ultrahigh thermal conductivity value, as presented before (Table 2).

An attempt was made to produce thermally conductive polymer composites by incorporating graphite nanoplatelets in bisphenol-A epoxy resin. In this process, the graphite nanoplatelets were functionalized in a two-step procedure, where methanesulfonic acid was used to functionalize, followed by the grafting of γ-glycidoxypropyltrimethoxysilane, and thereafter the polymer composite is prepared by casting. The results exhibited a thermal conductivity enhancement from the pristine bisphenol epoxy resin (0.201 W m^−1^ K^−1^) to 1.698 W m^−1^ K^−1^ with the addition of 30 wt% of functionalized graphite nanoplatelets [52]. Expanded graphite has been compressed to make a composite with a continuously expanded graphite network [53], potentially used in electronic cooling applications. The process included producing flaky multi plastic waste packing powder by using a solid-state shear milling followed by powder mixing. This established a good thermal conductivity through-plane (9.7 W m^−1^ K^−1^) and in-plane (10.1 W m^−1^ K^−1^) with an addition of 31.6 vol% expanded graphite [53]. Other researchers studied the influence of graphene fillers and hexagonal boron nitride fillers (hBNs) on the thermal conductivity of epoxy composites. They showed that the graphene fillers provide a better thermal conductivity improvement, which is explained by the higher intrinsic thermal conductivity of graphene filler. The highest thermal conductivity value was found to be approximately 11 W m^−1^ K^−1^ at 43 vol% and approximately 5.5 W m^−1^ K^−1^ at 45 vol% for graphene and hexagonal boron nitride fillers, respectively [54]. The usage of multiscale alumina particles to enhance the thermal conductivity of epoxy-based composite has also been reported. The thermal conductivity was improved from 0.28 W m^−1^ K^−1^ to 6.7 W m^−1^ K^−1^ upon the addition of 79 vol% of alumina particles with different sizes. When using a curing agent (5-Amino-1,3,3-trimethylcyclohexanemethylamine), a composite with a thermal conductivity value of 6.0 W m^−1^ K^−1^ was achieved. This lower thermal conductivity value can be attributed to the increase of interfacial resistance due to the gaps formed at the surface during curing [10].

In general, nanofillers have been blended with polymers to enhance their thermal conductivity, which has given positive results. However, researchers have been focused on finding new procedures to produce PMCs. An interesting method has been reported to improve the thermal conductivity of the polyphenylene-boron nitride composite, which involves the preparation of core-shell structure particles, creating a 3D segregated structure to enhance the formation of thermal networks and to improve thermal conductivity. This new approach has been compared to the polymer blending method, maintaining the 40 vol% boron nitride fillers, with enhanced thermal conductivity value (4.15 W m^−1^ K^−1^) when compared to the polymer melt blending approach (2.45 W m^−1^ K^−1^) [55]. A similar kind of approach was reported, with core-shell structured fillers being produced by the modification of boron nitride with styrene via polymerization. The composites produced with these fillers mixed with commercial polystyrene have achieved good thermal conductivity (0.692 W m^−1^ K^−1^) in comparison with the melt blending method (0.332 W m^−1^ K^−1^), when using ~15.9 wt% of boron nitride fillers [56]. A research study reported about 1700% improvement in thermal conductivity (4.79 W m^−1^ K^−1^) of a polyvinyl composite incorporated with 45.4 vol% alumina (Al_2_O_3_). The formation of a 3D thermal transport channel was the reason for this enhancement, where the composite was prepared using a vacuum-assisted infiltrating method [57].

A new method was reported based on the fabrication of polyethylene/hexagonal boron nitride composite sheets. It includes obtaining a multilayer structure (alternative high-density polyethylene filled with hexagonal boron nitride layers (5.67 vol%) and low-density polyethylene layers), annealed at 200 °C, which results in the diffusion of polyethylene molecules into the adjacent layers. This improves the formation of the thermal conduction network and enhances the thermal conductivity (1.37 W m^−1^ K^−1^) in comparison with the composite prepared with the same vol% by conventional methods (0.49 W m^−1^ K^−1^), which includes melt compounding using a twin-screw extruder [58].

The influence of the nanomaterial structure has been studied by the addition of two different fillers, namely copper nanowires and copper nanoparticles, to create a dimethicone nanocomposite. The addition of both fillers improved the thermal conductivity of the dimethicone matrix, which was 0.15 W m^−1^ K^−1^ [59], but the composites with copper nanowires (0.41 W m^−1^ K^−1^) reached larger values than the ones with copper nanoparticles (0.25 W m^−1^ K^−1^), when using 10% volume fractions. This phenomenon can be attributed to the large aspect ratio of copper nanowires, facilitating effective thermal networks, while copper nanoparticles were either small or encapsulated by a polymer, leading to poor thermal conductive pathways. An interesting approach (dual-assembly strategy) to produce polymer-graphene composites has been recently reported, showing a highly ordered graphene network with good contact between the adjacent graphene sheets. This method includes using polyurethane as a starting template to assemble graphene sheets, followed by performing pyrolysis of the graphene at 800 °C for one hour to remove the polyurethane template. The quality of the dual-assembled graphene framework is further improved by post-thermal annealing at 2800 °C. The vacuum infusion process was then adopted for producing an epoxy polymer composite [60]. The authors showed thermal conductivity values in the range of 56.8–62.4 W m^−1^ K^−1^ with the addition of 13.3 vol% of graphene, while epoxy-graphene composites have obtained a value of 62.4 W m^−1^ K^−1^. The authors reported that it could be the highest thermal conductivity ever achieved in graphene/polymer composites with the same level of graphene addition.

- Addition of more than one nanofiller

To improve the thermal conductivity at lower concentration levels, researchers have also focused on the addition of two or more nanofillers. The combined effect of two or more nanofillers when added simultaneously into a composite appeared as an attractive solution for the enhancement of thermal conductivity. Graphene nanoplatelets have been added to polycarbonate polymer, with the thermal conductivity reaching a maximum value of 1.13 W m^−1^ K^−1^ with 20 wt% GNPs, larger than the pristine resin, which was 0.24 Wm^−1^K^−1^. Thereafter, they attempted to replace a proportion of graphene with multi-walled carbon nanotubes and observed the synergetic effect of these two fillers, having raised the thermal conductivity to 1.39 Wm^−1^K^−1^ with a combination of 18 wt% GNP, 2 wt% CNT. This synergistic effect was due to the different sizes and shapes of fillers that have facilitated the formation of an efficient 3D thermal conductive network [61].

Wu and co-workers reported on the usage of MWCNT, along with expanded graphite (EG), in polypropylene nanocomposites for the improvement of electrical and thermal conductivity [62]. The formation of a double percolated filler network reduced the interface thermal resistance and facilitated the improvement of thermal conductivity. Regarding the single percolated networks, the thermal conductivity of the nanocomposite with expanded graphite was larger (0.9 W m^−1^ K^−1^) than the one with multi-walled carbon nanotubes (0.6 W m^−1^ K^−1^), when using 15 wt% of filler. However, the opposite was reported for the electrical conductivity, where PP/MWCNT composites and PP/EG composites presented a percolation threshold of about 2 wt% and 10–12 wt%, respectively. The ternary system of PP with MWCNT and EG, maintaining the 15 wt% EG above its electrical percolation threshold in PP, resulted in a sharp increase of both thermal and electrical conductivities to approximately 1.5 Wm^−1^K^−1^ and 10^2^ S.m^−1^, respectively, when using 5 wt% MWCNT content. The authors explain this synergetic effect when combining the two fillers by the expanded graphite as being two dimensional, with a more rigid structure that facilitated a reduction in interface thermal resistance, and multi-wall carbon nanotubes being one dimensional, with the thermal conductance limited to only radial direction [62].

A study was performed to investigate the synergetic effect of nanoparticles, namely boron nitride, graphene nanoplatelets, and short carbon fibers on the thermal conductivity of epoxy nanocomposites. At 3 wt% short carbon fibers + 5 wt% (boron nitride, surface modified graphene nanoplatelets), the achieved thermal conductivity was 0.8 Wm^−1^K^−1^, larger than the value for neat epoxy (~0.18 W m^−1^ K^−1^). This improvement was due to the created thermal channels between short carbon fibers and hybrid fillers, resulting in faster phonon conduction [63].

Recently, a novel method was reported to improve the thermal conductivity by conversion of high to low thermal dissipation of the thermal conductive network. In that study, polydimethylsiloxane was used as matrix, with short carbon fiber and glass bubble as the fillers. To convert high thermal dissipation to low thermal dissipation, two approaches have been employed, namely the densification of the thermal conductive network by spatial confining forced network assembly (SCFNA), and volume exclusion achieved by adding rigid particles. These approaches have successfully improved the thermal conductivity (11.423 W m^−1^ K^−1^), in comparison with the traditional compounding method (1.053 W m^−1^ K^−1^). Furthermore, the thermal conductivity was improved to 13.004 Wm^−1^K^−1^ with the addition of a 3 wt% glass bubble. This study proved that the optimal combination of these two approaches can improve thermal conductivity to a larger extent in comparison with traditional compounding methods [5]. Table 3 and Table 4 show the results of various researchers, achieved in an attempt to improve the thermal conductivity of PMCs by adopting several strategies.

#### 5.1.2. Microcomposites for Improved Thermal Conductivity

In ceramic fillers, heat will transfer due to phonons, caused by the lack of electrons, which explains why, in general, they present lower thermal conductivity values [16] (Table 2).

Polydimethylsiloxane (PDMS) was reinforced with carbon fibers (CF), prepared by a solution blending method and improving the thermal conductivity of the composite from 0.16 W m^−1^ K^−1^ up to 2.73 W m^−1^ K^−1^ upon the addition of 20 wt% CF. The improvement in the properties could be due to a decrease in the resistance between the CF and PDMS, owing to the overlapping of CFs. This led to the generation of an effective heat transfer path and hence an increase in thermal conductivity was observed [64]. A micro-phragmites communis structure was developed with CFs by using directional freezing, and they are reinforced with PDMS to enhance the through-plane thermal conductivity of the composites. The composite was prepared by using a vacuum-assisted method, having achieved a thermal conductivity value of 6.04 W m^−1^ K^−1^ at 12.8 vol% [35].

To investigate the thermal conductivity of PAN and pitch-based CFs reinforced ZrB2-SiC-ZrC (ZSC) matrix, a study was carried out by Inoue and co-workers. They found that pitch-based CFs have a larger thermal conductivity (45–70 W m^−1^ K^−1^) in comparison to PAN (8–12 W m^−1^ K^−1^) [36]. Strategies to obtain a 3D filler network were reported recently to improve the thermal conductivity of silicone rubber-alumina composite. The method involves foaming and a subsequent infiltrating process to produce a thermally conductive network framework. Ammonium bicarbonate was used as a foaming agent to produce the foam. The composite produced with this method has achieved a thermal conductivity value of 0.747 W m^−1^ K^−1^ at 32.6 wt% alumina, higher than the one achieved for the composite prepared with randomly dispersed alumina (~0.2 W m^−1^ K^−1^). This enhancement was due to an effective network, which created a strong interfacial bonding between the foam and the matrix [65].

Li et al. have also shown that the use of combined fillers can improve thermal conductance, in comparison with untreated carbon fiber fillers [66]. The spray drying method was used to attach spherical aluminum particles to the carbon fibers, reducing the interface thermal resistance of silicone rubber nanocomposites. The highest thermal conductivity (9.60 W m^−1^ K^−1^) was observed when 25 vol% of treated fillers were added. A designed structure was achieved through the addition of combined fillers, resulting in a reduced interface thermal resistance of composites. Additionally, hybrid fillers are composed of different sizes, and they complement each other to form a better thermal network, resulting in a thermal conductivity improvement [66].

#### 5.1.3. Multiscale Composites for Improved Thermal Conductivity

**Figure 5:**
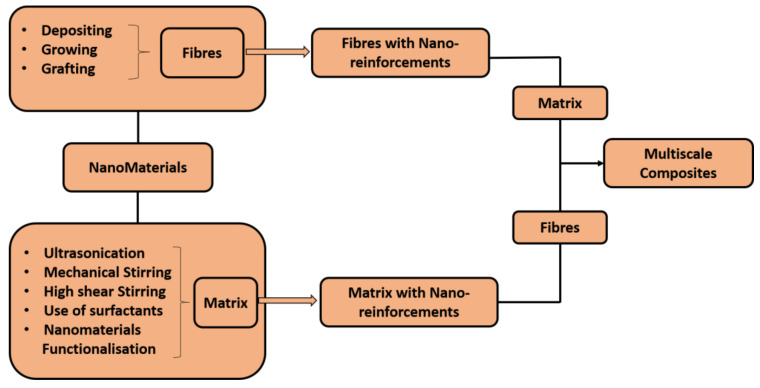
Schematic representation of various techniques to produce multiscale composites by two approaches.

Composites can be termed as multiscale composites when reinforcements at different scales (macro, micro, or nano) are added in the conventional composites to achieve new functionalities. These composites can be produced in two ways, which include the incorporation of nanomaterials into a fiber system or into a matrix system. The commonly used approach is the latter, the mixing of nano-reinforcements into a matrix. This approach includes the addition and mixing of nano-reinforcements into resins such as epoxy, phenolic, polyester, etc., followed by their application onto the fibers and subsequent cure or compression to produce a multiscale composite. One of the advantages of this route is its easiness and the ability for it to be applied to a wide range of nanomaterials. However, the major challenges are to achieve a uniform dispersion, since a non-homogeneous distribution can deteriorate the properties of the composites [67], and also to be able to achieve higher concentrations. Hence, to avoid the formation of agglomerations, physical and chemical techniques have been used, that go from ultrasonication, mechanical stirring and high shear stirring to the use of surfactants or nanomaterial functionalization. Several methods can also be used, such as depositing nanomaterials by electrophoresis, spraying, growing nanomaterials and grafting nanomaterials onto the reinforcements, where some of them involve high temperatures and require a long time [67]. The fabrication of multiscale composites using the two approaches of incorporating nanomaterials into a fiber system or into a matrix system are schematically represented in Figure 5. The most common methods of producing composites are compression molding, extrusion, injection molding, filament winding, resin transfer method, and vacuum infusion method. However, before this process, solvent casting, melt blending or in situ polymerization are employed to produce the intermediate products that are essential to produce composites [68].

A few works have been reported in the literature on the use of nanoparticles as secondary reinforcement to improve properties by introducing a hierarchical nature in the composites. Functionalization of nanofillers for their effective dispersion and successful deposition on the primary reinforcements has been used and reported, with this methodology (addition of a secondary reinforcement) ranking well for the improvement of mechanical properties. However, the usage of carbon-based fillers has additionally improved the thermal conductivity of the composites by reducing the interface thermal resistance and building good thermal networks. Some recent advancements are reviewed here and summarized in Table 4. CNTs have been used as a secondary reinforcement to improve the thermal conductivity of polyester/vinyl resin/glass fiber composites. The composites were prepared by injection method and the thermal conductivity was improved by 1.5 times when 3 wt% of CNTs were incorporated. Improvement of phonon diffusion with the addition of CNT could be the factor to improve the thermal conductivity of the multiscale composite [69]. Carbon nanofiber was used as nano-reinforcement to produce a multiscale composite where carbon fabrics were used as reinforcement in phenolic resins. The thermal conductivity was improved from 0.052 W m^−1^ K^−1^ to 0.071 W m^−1^ K^−1^ with the addition of 1.5 wt% of carbon nanofiber. Dispersion of carbon nanofibers might have formed links between the nearby carbon fibers and built a network that could contribute to the improvement of thermal conductivity [70]. Graphene foam was used in combination with carbon fiber in polydimethylsiloxane composites, and was found to enhance their thermal conductivity values. Upon the addition of 10 wt% carbon fibers, the thermal conductivity was raised to 0.39 W m^−1^ K^−1^ from the pure polydimethylsiloxane (0.21 W m^−1^ K^−1^). It was further enhanced to 0.55 W m^−1^ K^−1^ with the addition of graphene foam, in addition to carbon fiber. This improvement can be explained by the high thermal conductivity of graphene and its interconnected nature, the formation of a self-thermal conductive network of carbon fiber, and its connection with graphene to form an intensive network [11]. CNTs have been incorporated in the glass fiber Bisphenol-A epoxy composites, and a thermal conductivity improvement was visible with the addition of MWCNTs (0.47 W m^−1^ K^−1^, in comparison with the 0.38 W m^−1^ K^−1^ achieved for the composite without MWCNTs). The researchers also studied the influence of MWCNTs functionalization with γ-amino-propyltriethoxysilane (APS) on the thermal and mechanical properties of the composites. The addition of functionalized MWCNTs has furthermore improved the thermal conductivity (0.59 W m^−1^ K^−1^), in comparison to the other two cases. The reason for this improvement could be the formation of a better thermal conductive network owing to the better dispersion of functionalized MWCNTs [71]. CNTs were treated with H_2_SO_4_ under ultrasonic treatment and these treated CNTs were then used as secondary reinforcements to produce composites. Poly ether ether ketone (PEEK) was reinforced with carbon fibers (CF) and treated CNTs were sprayed to improve the thermal conductivity through thickness. The results exhibited a slight improvement upon the addition of 1.0 wt% of treated CNTs (1.15 W m^−1^ K^−1^) in comparison with PEEK-CF composites (0.97 W m^−1^ K^−1^). The improvement was claimed to be the improvement of phonon transport via well-connected thermal networks [72]. A synergistic effect was obtained when spherical Al_2_O_3_ was used in combination with GNPs in silicon rubber composites. When 9 wt% of Al_2_O_3_ was introduced in a silicon rubber matrix, the thermal conductivity of the composite reached the value of 2.29 W m^−1^ K^−1^ and the addition of 1 wt% of GNPs has improved this value to 3.37 W m^−1^ K^−1^. This significant growth was attributed to the 2D GNPs with an intrinsic higher thermal conductivity nature, which allowed for the creation of effective networks and decreased the thermal contact resistance due to high Al_2_O_3_ loadings [73].

A one-step deposition method for depositing copper and carbon nanotubes on carbon fibers was used, and its effect on the thermal conductivity of the composite was investigated. The authors used the vacuum bagging method to prepare the composite, and electrophoretic deposition was used to deposit copper and carbon nanotubes. They showed that the one-step method with positive charge CNTs gives composites with larger thermal conductivity values (2.948 W m^−1^ K^−1^) at 56.3 vol%, in comparison with the one-step method with negative charge CNTs (2.732 W m^−1^ K^−1^) at vol 56.4 vol%, and also compared to the two-step method (2.519 W m^−1^ K^−1^) at 56.9 vol%. However, both methods achieved an improvement when compared to epoxy-carbon fiber composites (0.642 W m^−1^ K^−1^). The thermal conduction network formed due to the addition of carbon nanotubes and copper nanoparticles facilitated phonon vibration and propagation, and, hence, the improvement of thermal conductivity [74]. The spraying method was adopted in another study to deposit GNPs onto the carbon fiber fabrics, and they are used as reinforcements in epoxy to fabricate multiscale composites by a vacuum-assisted resin transfer infusion (VARI) process. The deposition of GNPs reduced the thermal resistance between the matrix and the fibers and improved the thermal conductive networks, leading to a better phonon diffusion through thickness direction. In fact, the thermal conductivity of the composite was improved from 0.54 W m^−1^ K^−1^ (pristine composites) to 0.84 W m^−1^ K^−1^ with the addition of 0.5 wt% of GNPs [75]. CNTs were grafted onto the basalt fibers using polydopamine (PDA) as a coupling agent, and they were used as reinforcements in polyamide 6 (PA-6), with the fabricated composite showing improved thermal conductivity. The best value was obtained for the composites with the addition of PDA-CNT (~0.24 W m^−1^ K^−1^) in comparison with only basalt fibers (~0.19 W m^−1^ K^−1^). The slight improvement could be due to the enhanced interface bonding [76].

It has been previously reported that thermal conductivity depends strongly on the aspect ratio, length, size, diameter, and specific surface area of the filler. However, the methods of composite production and dispersion can also determine the achieved electrical and thermal conductivity.

### 5.2. Composites for Improved Electrical Conductivity

In addition, there has been a big effort on the production of composites to improve polymers’ electrical conductivities. As previously discussed, polymers are normally insulators, and thus there is a need to enhance their electrical conductivity for their use in a broad range of applications. Fillers with the ability to conduct electricity are added to polymers to improve their electrical conductivities [77]. They can also be considered nanocomposites or multiscale composites.

#### 5.2.1. Nanocomposites for Improved Electrical Conductivity

The intrinsic conductivity of the nanofillers has a strong impact on the maximum electrical conductivity of a nanocomposite. However, the electron loss at the junctions of the conductive network is also determinant. The latter is the reason why the overall electrical conductivity of a nanocomposite is usually 2–4 orders of magnitude lower than the intrinsic electrical conductivity of the nanofiller [48]. As previously mentioned, the delocalized electrons in graphite and graphene will facilitate the conduction of electricity. However, they have low electrical conductivity in the perpendicular direction, while graphene oxide presents a larger electrical conductivity of 6 × 10^3^ S/cm [78,79]. CNTs are one of the most suitable nanomaterials for improving electrical conductivity, with a value of 2 × 10^3^ S/cm. In turn, CNTs with longer length are known as carbon nanofibers, with larger electrical conductivities in the range of 10^5^ S/cm. Functionalization of CNTs has also improved the electrical conductivity of PMCs [78]. Graphite flakes present electrical conductivity values of up to 10^6^ S/m. Hence, many researchers have tried to incorporate them in various ways to improve the electrical conductivities of polymers. A few incorporated them in the matrix by mixing in resins or producing films with nanoparticles, and in some studies, they were sprayed on fabrics.

Graphene has been added (12 wt%) into a PDMS-urea copolymer and a film was produced with 1 mm thickness, showing an electrical conductivity of 81.5 S/m (see Table 5). The formation of stable conductive channels has facilitated the electrical conductivity in the films [80]. Cellulose nanofiber was used as a polymer and combined with reduced graphene oxide (rGO) to produce ultrathin flexible composite films by using a vacuum filtration process. The obtained films with 50 wt% of RGO obtained the highest electrical conductivity value of 4057.3 S/m, and can potentially be used as electromagnetic interference shielding materials [81]. Nanocomposites were produced with recycled polypropylene/polyaniline reinforced with graphene nanoplatelets using an ultrasonic-assisted screw extruder. These nanocomposites achieved an electrical conductivity of 4.1 × 10^−1^ S/cm with the addition of 3 parts per hundred resin (phr). These researchers reported the formation of a good network due to the addition of GNPs and the increase of contact between the GNPs and chains in co-polymer as the reasons to improve the electrical conductivity of the nanocomposite [82].

Hybrid nanofilms were produced by adding TEMPO cellulose nanofibrils (TOCNS) and carbon nanotubes in a pullulan matrix. These films’ electrical conductivity increases with the increase in CNTs, with an achieved value of 0.015 S/mm at 5 wt% of CNT and 0.5 *w/v* suspensions of TOCNS [83].

#### 5.2.2. Multiscale Composites for Improved Electrical Conductivity

Exfoliated graphene was spray-deposited on carbon fibers and they were reinforced with poly ether ether ketone (PEEK) to produce a composite. The introduction of the layer modified the electrical properties of the composite, which exhibited electrical conductivity values of 0.008 S/cm and 0.0037 S/cm in plane and through thickness, respectively [84]. The coating of graphene oxide (GO) with conducting polymers, namely polypyrrole (PPy) and poly(3,4-ethylene dioxythiophene) (PEDOT) was studied as additional reinforcements to carbon fabric in C-fabric-epoxy composites [85]. Coated graphene was introduced into the epoxy and deposited on carbon fabric, then stacking these fabrics to form a composite by maintaining 60% volume fractions. The addition of 0.5 wt% PPY/GO and 0.5 wt% PEDOT/GO has resulted in electrical conductivity values of 6.5 × 10^−3^ S/cm and 6.2 × 10^−3^ S/cm, respectively. Coating of conducting polymers might have improved the non-covalent interactions, which, in turn, increased the delocalization of charge and facilitated the movement of charge carriers. This explains the improvement of both systems in comparison with the electrical conductivity of C fabric-epoxy (0.42 10^−3^ S/cm) [85].

Three types of carbon nanomaterials, namely carbon blacks, flake graphite and carbon nanotubes, were used to improve the electrical properties of wood plastic composites [86]. A significant improvement in the electrical conductivity was observed upon the addition of nanomaterials, with decreased resistivity to the electricity flow with the increase in weight fractions. Among all of them, CNTs gave the best results, with the highest decrement in resistivity (6 × 10^5^ Ω.m) obtained for the composites with 12 wt% CNTs. This can be explained by the formation of a conductive network that reduced the resistivity of the composite [86].

### 5.3. Composites for Improved Thermal and Electrical Conductivity

Mxene/thermoplastic polyurethane multilayered flexible composite films were developed by using a layer-by-layer spraying technique to enhance both thermal and electrical conductivities. These films obtained an electrical conductivity value of 1600 S/m and in plane thermal conductivity of 6.31 W m^−1^ K^−1^. It was also reported to achieve 113 °C when a voltage of 5 V is applied for 10 s [87].

The influence of the aspect ratio on multiwall carbon nanotubes (MWCNTs) was investigated by producing epoxy/MWCNTs composites [88]. The results suggest that the addition of MWCNTs improved both the thermal and electrical conductivities of the composites, with a significant improvement in electrical conductivity and a moderate improvement in thermal conductivity. For the same 1 wt% of loadings, the composite that used higher aspect ratio MWCNTS presented a larger thermal conductivity value (~0.34 W m^−1^ K^−1^) than the composite with lower aspect ratio MWCNTs, which presented a relatively low value (~0.20 W m^−1^ K^−1^). This tendency can be explained by the assumption that the aspect ratio can influence the percolating network of nanomaterials in the polymer matrix. Additionally, similar observations were observed for the electrical conductivity.

Graphene was mixed with Epon 828, and carbon fabric was used as reinforcement to improve the thermal and electrical conductivities of the composite. With the addition of 1.0 wt% graphene, the thermal conductivity increased from 0.68 W m^−1^ K^−1^ to 0.72 W m^−1^ K^−1^, while the electrical conductivity improved from 5.6 × 10^−4^ S/m to 13.1 × 10^−4^ S/m [89]. In another study, graphene nanoplatelets were introduced into epoxy resin and resin was used to produce carbon fiber-reinforced plastics [90]. In-situ exfoliation with a three-way mill was used to add GNPs into epoxy, and a vacuum-assisted resin infusion method was used to produce the samples. The samples achieved 0.60 S/m and ~0.9 W m^−1^ K^−1^ for electrical conductivity and thermal conductivity, respectively, with the addition of 5 wt% GnPs. Polypropylene was reinforced with graphene nanoplatelets (different sizes) and the composites were produced with two different techniques: coating followed by compression molding and melt blending followed by extrusion [91]. The composites prepared by using the first method achieved larger electrical conductivity values than the ones produced by the second method (see more detail in Table 6). The coating is a better option, as the extrusion process did not ensure a proper exfoliation and dispersion of graphene into polypropylene.

Thermal conductive films were produced using graphene nanoflakes and these films were used to produce carbon fiber reinforced composites [92]. Unidirectional prepreg carbon fibers were used in this study, with the thermal conductive films placed on top and bottom, and cured in an autoclave oven to produce the composites. The electrical conductivity was reduced with the increase in thickness. Additionally, higher thermal conductivity (425 W m^−1^ K^−1^) and electrical conductivity (1799.45 S/cm) were achieved for the composites that do not have holes. The holes made in the film could have blocked the flow of electrons, which resulted in the reduction of electrical conductivity value.

An attempt was made to combine CNTs with GNPs and spray-coat them on carbon fibers to improve the electrical conductivity and thermal conductivity of the outer surface in woven fabric [93]. The surface resistivity of the samples was reduced by 2–3 Ω/sq with the addition of CNTs, and furthermore reduced to 3 × 10^−4^ Ω/sq with the addition of GNPs + CNTS. In turn, the thermal conductivity was improved from 200 W m^−1^ K^−1^ to 1500 W m^−1^ K^−1^.

The influence of Joule heating and thermal treatment on the electrical conductivity of phonelic resin samples reinforced with various weight fractions of carbon fiber, graphite and expanded graphite was investigated by Taherian and co-workers [94]. They showed that the electrical conductivity was increased for the first 60 s and then the slope of the curve decreased in almost all cases. The negative temperature coefficient of resistivity (NTCR) effect was observed, and, hence, Joule heating caused an increase in electrical conductivity in the samples. The thermal treatment shows both NTCR and the positive temperature coefficient of resistivity (PTCR) effect, where composites with graphite and carbon fibers showed the PTCR effect and composites with expanded graphite and combination of all nanomaterials showed the NTCR effect.

### 5.4. Methods to Produce Heating Composites

**Figure 6:**
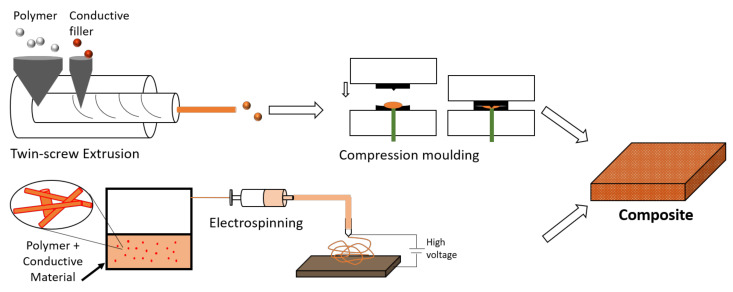
Schematic representation of two one-step techniques to produce a composite with heating capacity.

As already mentioned, there are several different ways of producing composites, which can largely affect their final thermal and electrical conductivities. The conventional methods include sol-gel method, blending method (either through powder, melting or solution), etc. The main issue of blending methods is the achievement of a proper filler dispersion within the polymer matrix. Since this dispersion is key to achieve an ideal electrical and thermal conductivity, a one-time in-situ polymerization becomes attractive for heating applications. This can be achieved through melt-mixing by a twin-screw extruder, followed by compression molding, or by electrospinning methods, as depicted in Figure 6.

Melt-mixing with a twin-screw extruder is a low-cost, eco-friendly method that presents the advantages of large-scale production and process easiness. The extrusion compounding process has been largely used for the preparation and processing of polymer composites. Its versatility allows for the tailoring of parameters for each material and application, which makes it even more promising for the preparation of conductive nanocomposites, by using carbon-based nanofillers, e.g., [95]. Polypropylene and graphene nanocomposites have been prepared using a co-rotating intermeshing twin-screw extruder by different researchers [91,96,97], with different exfoliation and dispersion levels when using different graphene nanoplatelets [98]. However, obtaining homogeneous dispersions within the polymer matrix is not an easy task, which is the reason why different techniques have been presented to achieve an improvement in the thermal, electrical and even mechanical properties of nanocomposites [95]. Silver/polypropylene nanocomposites have been prepared by extrusion after mixing and drying the silver nanoparticle solution and PP resins [99]. PP composites with MWCNT have been achieved by co-rotating twin-screw extruder and the obtained composites injection molded [100]. Other researchers have produced thermoplastic polypropylene/polyurethane composites with added graphene with a solution-flocculation and melt-mixing process using a twin-screw extruder [101]. Compression molding or injection molding is used afterwards to process the nanocomposites. The former has been shown to increase the electrical conductivity of PP/GnP nanocomposites, due to the formation of a continuous network of graphene [91].

Electrospinning is a very powerful technique for the fabrication of nanoscale continuous fibers. Through a fast and efficient process where an electrical field is employed, polymer solutions can form very fine 1D nanomaterials, with controllable diameters. Electrospinning is a recent low-cost, versatile and highly scalable fabrication method [102]. Different polymers can be used, such as polyurethane, polystyrene, or natural polymers. Polyaniline, and blends thereof, is the most used conductive polymer in electrospinning. Conductive polypyrrole nanofibers (diameters of about 70–300 nm) were also produced by electrospinning [103], and there are also some reported studies on PEDOT:PSS and polyimide. In turn, the use of electrospinning for the production of polymeric composites with nanofillers such as metal nanoparticles can be highly interesting, with nanofibrous mats as a promising carrier for nanofillers. In fact, the fillers’ dispersion is assured through this method and the contact between the adjacent fillers along the polymer fibers is increased, which subsequently improves the thermal conductivities [104]. The production via electrospinning has helped achieve the inclusion of thermally conductive fillers along an oriented radial direction of the obtained polymer fibers [104], improving the composite’s thermal conductivity. Since the percolation threshold for 1D sticks dramatically decreases as their length increases, extremely long nanofibers created by electrospinning can be highly promising for high-performance heaters. In a different approach, electrospun nanofibers of polyurethane were used as substrate for a subsequent coating of a hybrid material with silver nanoparticles and graphene nanosheets [105].

Some precautions should be adopted when producing composites if harmful or combustible solutions are used [106]. Metals, for example, can oxidize and become hydroxides in contact with ambient air, gaining unpredictable properties such as pyrophoricity. This oxidation can be avoided by using an inert gas, pre-drying or purifying the polymer.

## 6. Thin Film Heaters

Thin transparent conductive electrodes are highly needed for optoelectronic devices such as solar cells, organic light-emitting diodes (OLEDs), outdoor panels, displays, etc. [107]. Transparent conductive materials (TCMs) are used to produce these films, granting an acceptable sheet resistance and high optical transparency. The most employed TCMs are doped metal oxides, such as indium tin oxide (ITO), because it is conducting (10 Ω/sq) and highly transparent (~90%). However, ITO presents several drawbacks such as brittleness, high cost and high processing temperatures. In addition, there is a market growth in the TCMs, related with an increase in the need for flexible devices. These are the reasons why alternatives to ITO for flexible transparent thin electrodes have been sought in the latest years. These comprise the use of carbon nanotubes [108,109], graphene [110], silver nanowires [111] or nanocomposites of AgNW with PEDOT:PSS [112], or of AgNW with both CNT and PEDOT:PSS [113].

When a voltage is applied to a conducting film and it carries current, heat is produced through the Joule’s effect, acting as a heater, which is the focus of this review. ITO is also the most used TCM for transparent heaters, particularly for defrosting and defogging windows in vehicles, LCD panels and displays of avionics, etc. [114]. The era of metal oxide-based heaters included not only ITO but also fluorine-doped tin oxide (FTO) [115] and Al-doped zinc oxide (AZO) [116], in a combined effort to increase ITO thermal stability. The first transparent film heater using carbon nanotubes was reported by Yoon and co-workers [109], with a sheet resistance of 580 Ω/sq and a transmittance of 79% operable at 60 V. Since then, numerous studies have focused on different nanostructured materials for the next generation transparent thin heaters, which have been chronologically categorized in a comprehensive literature review [114].

### 6.1. Principles and Performance of Film Heaters

For a film heater, the electrical resistance R given by Equation (2) is now dependent on the cross-sectional area, given by the width W and thickness δ.

(4) $$R=\;\rho \frac{L}{A}=\rho \frac{L}{\delta W}$$

The sheet resistance R_S_, sometimes called sheet resistivity, is given by the resistivity ρ in thin films, which are uniform in thickness δ.

(5) $$R_{S}=\frac{\rho }{\delta }$$

Even though the material’s thermal conductivity determines the heat conduction, the film’s sheet resistance (R_S_) is what defines the heating rate and the steady state temperature attained [117]. For a material with certain electrical and thermal properties, the steady-state temperature increase is defined by the balance of Joule heating and heat dissipated by convection or radiation, and can be controlled by the voltage. In general, the lower the voltage required to reach a certain temperature rise, the better the heater. The key parameter for a good heater is its sheet resistance, with lower values giving rise to better power efficiencies. However, the sheet resistance uniformity is also critical to ensure an homogeneous temperature over the whole film. The film thickness, network density, area occupied by the material and % of conducting material can be varied to tune the transmittance and resistance of a given thin film heater. The power dissipated as heat depends on the thermal properties, not only the heating material, but also of the substrate where it is applied, and the interface also plays an important role [114]. Some heaters have a moderate thermal response due to the low adhesion to substrate, which causes nonefficient heat transfer and nonuniform temperatures.

**Figure 7:**
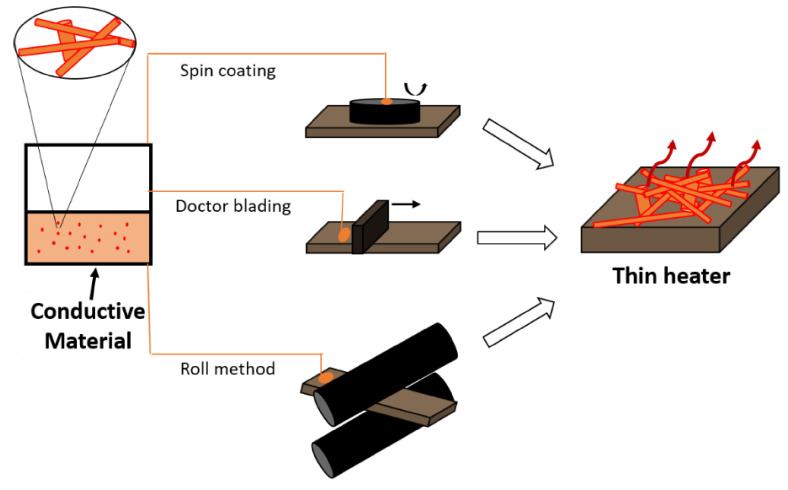
Schematic representation of some of the methods to produce a thin film heater from a conductive material solution.

The performance comparison of heaters using different materials is not an easy task since a well-established Figure of Merit (FoM) is normally not reported by the researchers. To allow for an easier comparison and benchmarking, Sorel et al. developed a comprehensive theoretical framework that relates temperature increase, transparency, electrical and thermal properties [117]. Thin film heaters can be prepared by numerous methods, with only some of them schematically represented in Figure 7.

### 6.2. Carbon-Based Heaters

Even though SWCNT transparent heaters perform better than ITO films, their dispersion requires surfactants and strong acid treatment, compromising the conductivity of SWCNT-based films. After the first report using SWCNT for transparent thin heaters [109], others appeared using multi-walled carbon nanotubes (MWCNT) [108], performing slightly better in terms of input power and flexibility. However, graphene then appeared as a highly promising heating element, since its ultrahigh thermal conductivities would result in uniform temperature distribution and fast heating rates [118]. It has been reported that defects and grain boundaries, as well as a high contact resistance between graphene flakes, can explain the need for a high applied voltage. Additionally, when the thickness of the graphene film is increased (which is needed in order to lower the R_S_), the transparency is decreased, so a balance between conductivity and transparency needs to be achieved. Instead of increasing the film thickness and compromising the transmittance, another solution to improve the heater performance is doping. Kang et al. [119] used two wet chemical dopants, gold chloride nitromethane AuCl_3_-CH_3_NO_2_ and nitric acid HNO_3_, to lower the sheet resistance of graphene films. They showed that graphene heaters doped with AuCl_3_ exhibited the best performance with higher steady state temperature up to 100 °C at 12 V, as opposed to the 65 °C saturation temperature for HNO_3_-doped graphene heaters (see Table 7 for details). Bae et al. showed that the use of a roll-to-roll chemical vapor deposition (CVD) process to produce defect-free graphene on copper substrates is highly promising. They also reported that layer-by-layer stacking, multiple transfer and doping with wet chemical p-dopants can enhance the electrical and optical properties for transparent electrodes, reporting a R_S_ of ~30 Ω/sq with ~90% optical transmittance, which is superior to commercial ITO-based electrodes, for p-doped four-layer graphene film [120]. A full analysis of the heat dissipation through convection is performed when CVD-grown large area graphene on copper foil is used as defogger [121]. A trilayer of graphene doped with AuCl_3_ achieved a sheet resistance of 66 Ω/sq with ~90% optical transmittance with outstanding heating performance, as presented in Table 7.

### 6.3. Metal Nanostructure-Based Heaters

Bulk metals reflect in the visible range, requiring an extremely small thickness to ensure transparency. However, as film thickness decreases from 10 nm, a dramatic sheet resistance increase occurs, which is due to electron scattering because of substrate and grain boundaries [122]. Metal nanostructures can thus be employed to create thin films, by manipulating photons and electrons to achieve the desired properties. Metal nanogrids have been shown to present good heating performance, with the line width of the metal mesh and the period of the mesh having a strong impact on the transparency and uniformity of current, respectively [123]. However, the costly and complex fabrication process of nanogrid-based transparent electrodes, which requires vacuum deposition, renders them far from ideal for real application, and opens the quest for alternative solutions.

Metal nanowires, randomly distributed in networks, appeared as an emerging candidate for TCMs, as they are solution-processable nanomaterials, where large area and low-cost deposition techniques are possible. Metal nanowires with percolating networks combine high electrical conductivity, optical transparency and flexibility [124], which makes them a highly promising alternative. In addition, much lower raw material quantities are needed to achieve the same electrical resistance and optical transparency of an ITO-based electrode, owing to the high aspect ratio of metal nanowires. The geometries of metal nanowire networks are similar to those of CNT, as they organize themselves forming cross-bar junctions. As previously stated, silver nanowires probably rate as the best candidates for transparent heaters since they present a very large thermal conductivity (thanks to silver). In addition, the low sheet resistance enables AgNW heaters to reach higher temperatures at lower voltages. The fabrication and usage of silver nanowires for flexible transparent heaters have been extensively reviewed [125]. Celle and co-workers [126] reported excellent heating performances for AgNW transparent heaters, by spin-coating or spray-coating random AgNW networks on glass or plastic substrates (see Table 7). They showed that the heaters reached a temperature of 55 °C under an applied voltage of only 7 V. Moreover, the quantity of AgNW has been measured and tens of mg·m^−2^ were enough to reach well-performing thin film heaters [111,126]. The properties of the silver nanowire networks, and consequently the performance of the AgNW-based thin film heaters, depend greatly on the properties of the fibers given by the synthesis (diameters, functional groups, etc.), on their interconnections (wire junctions) and on the network density [124]. In fact, the percolation-like conductivity of these nanowires makes it possible to tune the film’s sheet resistance while maintaining a relatively high total transmittance. It has been indicated that smaller diameters and larger nanowire lengths give better-performing heaters.

However, the breakdown of silver nanowires under high currents is a known issue [127,128]. This is explained by a relatively high nanowire–nanowire junction resistance and is intimately linked with the formation of hotspots due to self-aggregation of wires. Since these problems must be avoided at all costs in thin heaters, some solutions for this problem have been explored throughout the years, with the final objective of increasing electrical conductivity. Unlike carbon-based materials [129], metal junctions can be sintered to reduce junction resistance. Thus, these solutions include annealing, self-joining of network junctions by post-treatment or interconnecting the NWs with other conducting materials. This is why the fabrication of interconnected AgNW networks has been carefully studied [114,124]. This issue has been addressed by Kim et al. [130] by dispersing the AgNW with clay platelets prior to deposition.

### 6.4. Conductive Polymer-Based Heaters

Even though there were indications that intrinsically conductive polymers could have excellent electrical conductivity, the first 100% polymeric thin heaters were reported by Gueye et al. in 2017 [131]. Four (PEDOT)-based transparent thin films have been studied, with PEDOT:Sulf showing to have the best heating properties, as presented in Table 7. Even though the commercially available PEDOT:PSS presents lower conductivities, due to the presence of insulating PSS, the ethylene glycol-treated PEDOT:PSS produced excellent performance heaters, with 68 Ω/sq at 89.6% transmittance. In fact, the Figure of Merit, calculated by the ratio between DC and optical conductivity, proves that PEDOT:PSS-EG and PEDOT:Sulf have equivalent heating properties. PEDOT-based thin heaters are very competitive with other state-of-the-art nanomaterial-based heaters, performing better than metal oxides, CNT and graphene, and almost as well as metal nanostructures and derived hybrids. The easiness of thin heater production by spin-coating solution-processable PEDOT-based materials on top of glass, flexible PET or polycarbonate substrates open up a range of new possibilities for devices. However, the possibility of PEDOT:PSS to degrade at high temperatures limits its use for some applications.

### 6.5. Nanocomposites and Hybrid Heaters

The use of different materials is highly promising for producing heaters, with the combination of their individual advantages resulting in better performances than those achieved when using a single material. The previously discussed composites with high conductivity can be used in the development of thin heaters, using different production methods. Coatings with protective layers can additionally be interesting to offer higher electrical and thermal stability [132].

Lowering the overall R_S_ and securing a homogeneous heating is made possible by using a synergetic interplay between AgNW networks, for example, and an additional entity. Several ways of decreasing nanowire junction resistance, avoiding hotspots and assuring a uniform temperature distribution in AgNW-based heaters, have been explored and reviewed [114]. Efficient, low power hybrid heaters can thus be designed when following different strategies:

- Add another percolating network using CNTs [133] or graphene flakes [134];
- Coat with thin film of conducting polymers [135,136];
- Embed the nanowire network in an insulating polymer [137,138].

Single-walled CNTs have been used as networking material to improve the stability of AgNW networks under current flow. In order to avoid dispersion agents or surfactants, which would require an extra step of removal, Woo et al. demonstrated a method of directly mixing SWCNT functionalized with quadruple hydrogen bonding (QHB) motifs with an aqueous suspension of AgNW [133]. In turn, Zhang and co-workers used graphene oxide microsheets as an over-coating to protect the AgNW network [134]. This is performed not only to avoid coalescence into isolated Ag particles, but also to avoid local oxidation of AgNW, leading to an abrupt rise in sheet resistance. They showed well-performing thin film heaters following this architecture (see Table 7). Another kind of hybrid material was recently reported by Choi and colleagues, producing a transparent heating film by coating a solution of graphene oxide and silver nanowires on PET or glass [139].

Spin-coating of PEDOT:PSS on top of AgNW network has been shown by Li et al. to fill the voids and reduce the interwire contact resistance [136], as previously pointed to in this review as a problem. Apart from helping the adhesion of the network to the PET substrate, due to the downward force, this method improved the conductivity values without changing the transmittance. The authors explain the drop in contact resistance by the difference in work functions between PEDOT:PSS, a p-type semiconductor, and AgNW, which induces electronic transmission channels [136]. A detailed thermodynamic analysis has been performed in order to give a guidance on the design of transparent heaters with tunable response time for different application queries [135]. Ji et al. focused on hybrid AgNW/PEDOT:PSS transparent film heaters on glass and PET substrates with different thicknesses to evaluate their impact over thermal properties from the heat capacity point of view [135].

Heat-resistant polymers, instead of thermally degradable PEDOT:PSS, have been used to bond to the AgNW network, allowing for high temperatures at low operation voltages [137]. Li et al. produced a transparent composite film of highly conductive AgNW and a polyacrylate-based heat-resistant insulating polymer matrix, showing very promising heating performances [137], comparable to the ones achieved for AgNW/PEDOT:PSS [136] (Table 7). Li and co-workers reported a method of promoting strong bonding between the thin layer of AgNW conductive network and the polymer substrate [137].

Additionally, Entifar and co-workers [140] showed an improvement in transparent films of silver nanowires when elastomeric substrates were treated with 11-aminoundecanoic acid, and also coated with PEDOT:PSS. Another study used polydimethylsiloxane (PDMS) as a stretchable substrate, and showed great electrical and thermal conductivity at high optical transmittance, alongside the superior mechanical properties [141]. In fact, they developed a full study on the stretching effect on the heating performance, concluding that the AgNW/PDMS electrode is a stretchable transparent heater with satisfactory properties to be applied in future wearable electronics.

A comparison between all nanomaterials used in the next-generation thin film heaters is gathered in Table 7.

After a detailed analysis, it becomes clear that hybrid materials are the best-performing thin film heaters. Specifically, silver nanowires with PEDOT:PSS rate as the most promising composite candidates, making efficient and flexible film heaters with the lowest sheet resistance. The PEDOT:PSS coating, which lessens the nanowire junction resistance but also enhances the adhesion to the substrate, is key for the efficient heat production by the Joule effect. However, the maximum temperature that they can reach is limited by the relatively low thermal resistance of the transparent polymer substrate. The production of designed composites to make thin heaters is thus a possibility for enhancing the properties. This indicates that the combination of multiscale materials can be highly advantageous for heating technologies.

## 7. Heating Textiles

**Figure 8:**
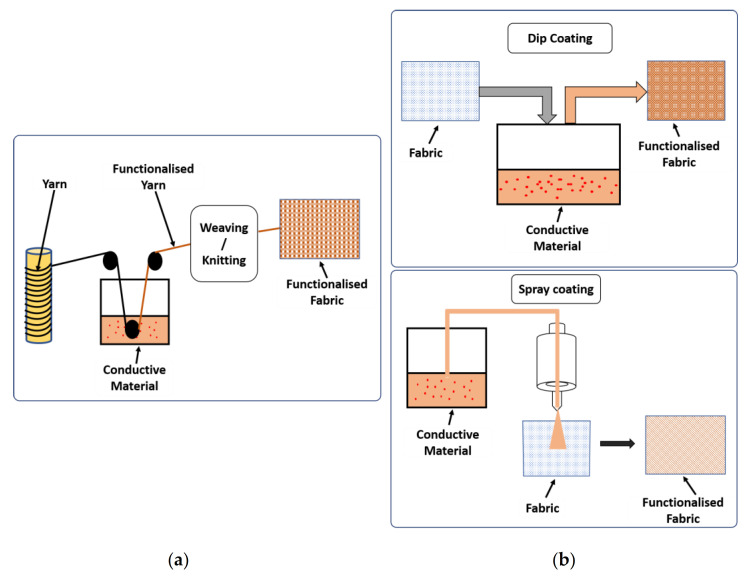
Schematic of the different processes to obtain a conductive fabric: (**a**) Functionalization of the yarn to be used in weaving/knitting to fabricate a conductive fabric; (**b**) Functionalization of a conventional textile fabric, through dip-dry coating or spray coating techniques with a conductive material.

Wearable electronics have been of growing interest in the latest years, in order to improve the quality of people’s lives by monitoring physiological and environmental parameters of the human body. The unique characteristics of textiles such as light weight and flexibility make textile-based wearable electronics highly promising [142]. In addition to electronic and smart textiles, personal thermal management has also appeared as a desired property in thermoregulating textiles [143]. In fact, they are useful in the household, for technical purposes, and in the automotive or medical fields [144]. Even though heating textiles can be based on solar heating, chemical heating and phase change material heating [145], this review focuses on electric heating, taking advantage of the Joule effect. For this, a conductive material must be used within the fabric to generate heat when an electric current is applied, since commonly used textiles such as cotton, nylon and polyester are insulators. The electric heating element must be composed of a conductive material, which can be metallic or non-metallic, and the substrate material, which defines the flexibility, stability and safety of the textile [145]. Metallic wires, commonly found in electric blankets, have been used at least since World War II in textiles [144]. However, they present several drawbacks, such as stiffness and brittleness. Thus, there has been some evolution to produce more comfortable integrated solutions for different applications. Heating textiles can be divided into two categories depending on their processing method: one is to fabricate conductive yarns and to use them on the fabrication of a textile with the same features through weaving/knitting. The other one is to functionalize a non-conductive textile fabric through coating (Figure 8).

### 7.1. Conductive Yarn-Based Textiles

Conductive yarns have been researched and developed for heating textiles [37]. Intrinsically conductive yarns composed of conductive polymers or metals, such as stainless steel yarns, are the most common for producing a conductive fabric. However, hybrid yarns, such as metal-coated polymer yarns, present advantages for the combination of a conductive and a resistive element for energy release in the form of heat [146]. Shahzad et al. used polyester to offer more resistance towards the electric current, and stainless steel to offer higher electrical conductance, showing the effect on heating behavior of a hybrid spun yarn [146]. Other authors have shown the potential of a tri-component elastic-conductive composite yarn, t-ECCY, which is composed of elastane filament, stainless steel filament and rayon fibers [147]. They proved that commercial knits decorated with a single t-ECCY performed well, with rapid thermal responses and uniform surface temperature distributions, easily controlled by the applied voltage [148].

As opposed to conductive textiles with sewed or embroidered solid metallic wires, conductive yarn-based textiles can be produced to attain the properties of non-conductive textiles. In fact, different parameters can be tuned, taking into account the necessary conductivity, durability and comfort [37]. Among them are the conductive yarn material, the method to be used during yarn processing, the proportions between conductive and non-conductive yarns and the distribution of the conductive yarns in the structure.

Other available yarns are non-conductive common yarns functionalized in a way that makes them electrically conductive. There has been some research regarding the use of nanomaterials for creating functionalized yarns. There are some companies supplying common yarns with graphene, which can be interesting for several antimicrobial or thermal-regulating applications, but not for heating textiles, as they do not present electrical conductivity. Coating with a metal film, usually silver, is another possibility. For instance, there is a study reporting the coating of nylon, polyester and cotton yarns with a metallic mesh made of random networks of AgNW [149]. The authors showed that these threads were turned electrically conductive by simply dip-coating them in a AgNW solution, with less resistance yarns formed when using larger AgNW densities. Atwa et al. demonstrated that thread heaters were formed by increasing the temperature above 50 °C at a bias of 1.5 V [149]. Even though silver is expensive, this AgNW mesh coating spends less than 5% of the typical 1 µm-thick silver. Yang et al. have produced a yarn of PET, silver nanowires and polydimethylsiloxane (PDMS), which presents high electrical conductivity (3 Ω cm^−1^) [150]. They have proven that an increase in drop-casting cycles produces a better AgNW coating, resulting in a decrease in resistance due to a better wire interconnection. Additionally, Hwang et al. demonstrated the production of a highly conductive machine-washable yarn made of a silk yarn dip-coated with AgNW and then coated with PEDOT:PSS, [151]. They proved the usability of this conductive yarn by fabricating a woven heating fabric with it and producing enough heat at low voltages through the Joule effect. The AgNW coated with the inherently conductive polymer gives rise to a conductive composite-coated yarn that is used as weft in a plain weave textile [151].

When comparing heating elements made of nonwoven fabric, woven fabric and knitted fabric, the woven is the best performing one, followed by knitted fabric and nonwoven fabric. This is dependent on the structure of the materials, with conductive nonwoven fabric presenting high electrical resistance [144]. Hamdani et al. studied different features of fabrics knitted with silver yarn and elastomeric yarn for heating textiles [144]. They used plain, rib and interlocked structures with silver yarn and showed that the interlocked structure had the best heating performance. However, and since the latter allowed more current to pass through at relatively lower voltages, the interlocked structure suffered degradation before the other structures [144]. In turn, Hao et al. used silver filaments or coated silver yarns for weaving into plain fabrics [152]. They reported a promising method of fabricating flexible heating textiles with intermittent conductive and non-conductive yarns, which allows for a tighter control over the density of conductive filaments and power optimization.

### 7.2. Functionalized Textile Fabrics

The modification of conventional textiles with a conductive solution can be performed by casting, depositing, spinning, printing, solution growth or dip-and-dry methods. Among all of them, the dip-and-dry method is the easiest and most cost-effective. Coating of standard textiles with conductive materials gives rise to a higher temperature homogeneity than with the yarn-based textiles. This is because the latter relies on localized heat production at the conductive yarns [118]. In addition to maximizing heat production and minimizing response time (see Table 8), eliminating hot spots is another crucial requirement.

Carbon nanotubes have been used as a conductive material in heating textiles [153,154]. Rahman and co-workers functionalized cotton fabrics with a MWCNT by using a simple dip-dry coating technique [154]. Even though the thermal conductivity of the cotton fabric was increased from 0.027 W m^−1^ K^−1^ to 0.045 W m^−1^ K^−1^ upon functionalization with MWCNT and the achieved temperatures meet the demand (see Table 8), the resulting heating textiles require high operating voltages of 40–60 V [154]. This is believed to happen because of the high contact resistance in CNT, which requires extensive power consumption, making them inappropriate for e-textiles. When coating a knitted cotton fabric with SWCNT through a dip-dry method, Yang et al. showed promising bending and thermal properties for application in wearable electronics [155].

Silver nanowires have been used to coat different fabrics using the dip-and-dry method [149,156]. The electrical conductivity and heating properties place AgNW-coated textiles as very promising heaters, which was also studied under mechanical treatments. Doganay et al. have performed an extensive study [156], proving that each dip-dry cycle increased the number of deposited AgNW on the cotton fabrics and consequently decreased the sheet resistance (2.5 Ω/sq after 25 cycles). Additionally, AgNW networks have been shown to possess infrared reflection properties, with passive insulation adding up to Joule heating for personal thermal regulation [157].

An inherently conductive polymer PEDOT:PSS was used for the first time with an anionic surfactant SDS to produce stretchable fabric heaters [158]. Yeon et al. performed an extensive study on coating methods and showed that the highest conductivity was achieved when blending was followed by dipping methods in the fabrics. They showed promising results for heating textiles (see Table 8).

Combinations of carbon-based materials with other polymers to give composites is also explored for coating fabrics. Tian et al. developed a graphene/polyurethane composite ink and created a bilayer structure by spray-coating the ink on top of a cotton fabric substrate [159]. They have shown promising Joule effect fabric heaters with a high steady-state temperature of 162.6 °C and a fast heating rate (8.4 °C/s) under 12 V. Another study showed the coating of cotton fabrics with reduced graphene oxide, followed by the incorporation of Ag and Cu nanoparticles [160]. They proved that Cu NP incorporation reduced the surface resistivity and thus increased the Joule heating electrothermal property. Very recently, a cotton textile was dip-coated with an electroconductive composite of reduced graphene oxide and PEDOT:PSS [161]. Ahmed and co-workers showed the beneficial use of an inherently conductive polymer for coating the voids of textile fabric and thus increasing the electrical conductivity. They studied different conditions of coating, having achieved the lowest sheet resistance (presented in Table 8) when the fabric was first dipped into rGO once, followed by dipping into PEDOT:PSS suspension 15 times [161].

Heating patches can also be added to a specific place of a textile through sewing. These can be made by combining heating materials and a carrier within bus bars to supply the power.

## 8. Applications

Joule heating is used in multiple applications such as immersion heaters and water boilers, but also carbon fiber-reinforced plastics [162]. Functional fibers, composites and textiles using the principle of the Joule effect are used in warming and therapeutic applications [1]. The production of fabrics that actively produce heat, reaching a controlled temperature by the application of a small voltage, is highly promising for personal thermal management [163]. In addition, smart textiles and wearable electronics are also possible applications [164], with Joule heating as a promising approach for the manufacturing of flexible and large-area sensors and electronic devices [165]. Heating composites can be used in self-reparation [166], healing [167] and de-icing applications [168]. Furthermore, heating fabrics and composite films are finding their ways in biomedical and cosmetic applications, with benefits for wound healing [169].

**Figure 9:**
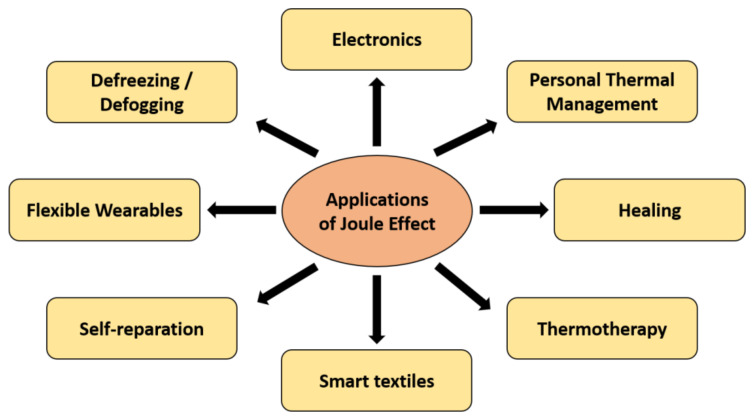
Applications of systems using the Joule effect in various sectors.

Transparent heaters developed on the principle of Joule heating can be used in defogging/defrosting windows [1]. In fact, their first application was on aircraft windshields to avoid condensation and increase visibility during military actions [132]. Thus, transparent heaters must meet the requirement to sustain harsh heating conditions in military applications [114]. These thin film heaters can also be applied in flexible solar cells, displays such as organic light emitting diodes (OLEDs) or touch panels [125]. Thermochromic devices and medical applications are also possible for thin heaters. The different applications of Joule heating systems, which can be used in different assemblies, are shown in Figure 9.

**Figure 10:**
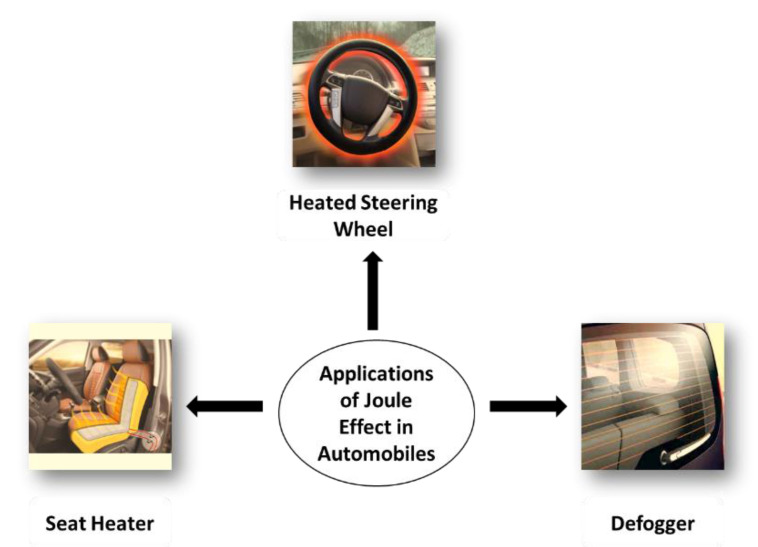
Automobile applications using the Joule effect.

In particular, applications related to the automotive sector are shown in Figure 10. In addition to the previously mentioned defoggers, Joule effect-based materials can also be applied as seat and steering wheel heaters [162].

## 9. Conclusive Remarks and Prospects

In this review, we have discussed the heat transfer mechanisms, including thermal conduction in crystalline materials and polymers. Different emerging intrinsically conductive materials have appeared lately, with carbon-based nanomaterials and silver nanowires as the most promising conductive nanofillers for nanocomposites. These nanomaterials can be used on their own or combined with polymers in a composite to improve the thermal and/or electrical conductivity of the latter. There has been a large amount of research on the development of heaters through the Joule effect in recent years. These nanomaterials can be used within fibrous or composite systems and structured from the nano up to the macroscale to produce heating applications such as thin film heaters or heating textiles. This review thus presents a multiscale approach for the production of heaters that can rely on different scale conductive materials or on material interactions across scales. In addition, different methods are reviewed on the way to produce these composites, thin film heaters or heating textiles. Since meeting all industrial requirements with a single material is nearly impossible, the combination of several materials can present enhanced properties or better stability. To this extent, this review could be highly important in guiding on the exploitation of specific materials for the development of a heating technology that can be applied to industry standards in the near future.

## Figures and Tables

**Table 1:** Thermal conductivities of generally used polymers [3,9,10,11].

Polymer	Thermal Conductivity (W m^−1^ K^−1^)
Acrylonitrile butadiene styrene (ABS)	0.18
Epoxy resin	0.20–0.88
Low-density polyethylene	0.32–0.40
High-density polyethylene	0.38–0.51
Ultrahigh molecular weight polyethylene	0.41–0.51
Nylon 6	0.29
Nylon 6.6	0.23
Polybutylene terephthalate	0.21
Polydimethylsiloxane	0.19–0.21
Polyetheretherketone	0.25
Polyethylene terephthalate	0.24
Polyimide	0.29–0.35
Polymethylmethacrylate	0.18
Polypropylene	0.17–0.22
Polyphenylene sulfide	0.25
Polystyrene	0.18
Polysulfone	0.28
Polytetrafluoroethylene	0.25
Polybutylene terephthalate	0.25–0.29
Polyvinyl chloride	0.14–0.17
Polyvinylidene chloride	0.13

**Table 2:** Thermal conductivities of generally used materials.

Materials	Category	Thermal Conductivity (W m^−1^ K^−1^)	Ref.
Carbon fiber	Carbon-based	400–650	[14]
Carbon nanotubes	Carbon-based	3000	[14]
Graphite	Carbon-based	100–400	[15]
Graphene nanoplatelets	Carbon-based	2000–6000	[9]
Graphene sheets	Carbon-based	~5000	[16]
Single-walled carbon nanotubes	Carbon-based	2000	[16]
Multi-walled carbon nanotubes	Carbon-based	3000	[16]
Aluminum	Metal	247	[17]
Copper	Metal	398	[17]
Gold	Metal	315	[17]
Silver	Metal	427	[17]
Silver nanowires	Metal nanostructure	191.5	[18]
Polyacetylene	Conductive polymer	0.4–13	[19]
Polypyrrole (PPy)	Conductive polymer	0.11–0.25	[19]
Polyaniline (PANi)	Conductive polymer	0.04–0.14	[19]
Polythiophene	Conductive polymer	0.2–4.4	[19]
Poly(3,4-ethylenedioxythiophene):Polystyrenesulfonate (PEDOT:PSS)	Conductive polymer	0.16–0.39	[19]
Diamond	Ceramics	1000	[3]
Aluminum nitride	Ceramics	100–300	[9]
Beryllium oxide	Ceramics	230–330	[20]
Cubic boron nitride	Ceramics	1000	[21]
Hexagonal boron nitride	Ceramics	300	[21]
Silicon carbide	Ceramics	120	[22]
Aluminum oxide (α-Alumina)	Ceramics	30	[3]

**Table 3:** Thermal conductivities obtained with the addition of various reinforcements at different scales.

Polymer	Fillers	Filler (%)	Thermal Conductivity (W m^−1^ K^−1^)	Additional Information	Ref.
E 51	GNPs	30 wt	1.70	Functionalized GNPs	[52]
MPW	EG	31.6 v_f_	10.1	In Plane	[53]
MPW	EG	31.6 v_f_	9.7	Through Plane	[53]
PPH	hBN	40 v_f_	2.45	Melt blending	[55]
PPH	hBN	40 v_f_	4.15	Core shell structure nanoparticles	[55]
CPS	BN	~15.9 wt	0.33	Melt blending	[56]
CPS	BN	~15.9 wt	0.69	Core shell structure nanoparticles	[56]
PVA	Alumina	45.4 v_f_	4.79	Vacuum-assisted infiltrating method	[57]
PE	hBN	5.67 v_f_	0.49	Conventional method	[58]
PE	hBN	5.67 v_f_	1.37	Multilayered structure through annealing	[58]
Epoxy	Graphene	43 v_f_	11	High shear speed mixer and vacuum chamber	[54]
Epoxy	hBN	45 v_f_	5.5	High shear speed mixer and vacuum chamber	[54]
DMC	CuNPs	10 v_f_	0.25	-	[59]
DMC	CuNWs	10 v_f_	0.41	-	[59]
S R	Alumina	36 wt	0.2	Dispersed randomly	[65]
S R	Alumina	36 wt	0.75	Foaming creating 3D network	[65]
PCL	GNPs	20 wt	1.13	One filler	[61]
PCL	GNPs + MWCNTs	18 wt, 2 wt	1.39	Two fillers	[61]
PP	MWCNTs	15 wt	0.6	-	[62]
PP	EG + MWCNTs	15, 5 wt	1.5	-	[62]
Epoxy	SCF, BN + mGNPs	3, 3 wt	0.56	Surface modified GNPS	[63]
Epoxy	SCF, BN + mGNPs	3, 5 wt	0.8	Surface modified GNPS	[63]
SR	Alumina + CF	25 v_f_	9.60	-	[66]
PDMS	SCF	30 wt	1.05	TCA	[5]
PDMS	SCF	30 wt	11.42	SCNFA	[5]
PDMS	SCF+GB	30+3 wt	13.00	SCNFA+VE	[5]
Epoxy	Alumina	79 v_f_	6.7	Multiscale Fillers	[10]
Epoxy	Alumina	79 v_f_	6.0	Multiscale Fillers and curing agent	[10]
Epoxy	Graphene	13.3 v_f_	62.4	Dual assembled graphene framework	[60]

BN, boron nitride; CPS, commercial polystyrene; CF, carbon fibers; CuNPs, copper nanoparticles; CuNWs, copper nanowires; DMC, dimethicone; E 51, bisphenol-A epoxy resin; EG, expanded graphite; ES, electrical stirrer; GB, glass bubble; GNPs, graphene nanoplatelets; mGNPs, surface-modified graphene nanoplatelets; hBN, hexagonal boron nitride; MG, mechanical grinding; MPW, multi-plastic waste; MS, mechanical stirring; MWCNTs, multi-wall carbon nanotubes; PCL, polycarbonate; PDMS, polydimethylsiloxane; PE, polyethylene; PP, polypropylene; PPH, polyphenylene; SCF, short carbon fibers; SCNFA, spatial confining forced network assembly; SR, silicone rubber; TCA, traditional compounding method; VE, volume exclusion; v_f_, volume fraction; wt, weight fraction.

**Table 4:** Thermal conductivities of multiscale PMCs.

Polymer	Fillers	Filler (%)	Thermal Conductivity (Wm^−1^K^−1^)	Additional Information	Ref.
PDMS	CF	10 wt	0.39	High speed shearing and stirring method	[11]
PDMS	CF+GF	10 wt, 0.5 wt	0.55	High-speed shearing and stirring method	[11]
Phenolic resin	CF	41 v_f_	0.052	No CNFs	[70]
Phenolic resin	CF + CNFs	41 v_f_, 1.5 wt	0.071	1.5 wt% addition of CNFs	[70]
Bisphenol-A epoxy	GFs	N/A	0.38	Without MWCNTs	[71]
Bisphenol-A epoxy	GFs + MWCNTs	0.5 g/L	0.47	Ultrasound-assisted impregnation method to deposit MWCNTs on GFs	[71]
Bisphenol-A epoxy	GFs + MWCNTs	0.5 g/L	0.59	MWCNTs functionalized with γ-amino-propyltriethoxysilane (0.025 g/L)	[71]
PEEK	CF	N/A,	0.97	No CNTs	[72]
PEEK	CF + tCNTs	N/A, 1.0 wt	1.15	Treated CNTs, spraying method, CM	[72]
SR	SAl_2_0_3_	N/A, 90 wt	2.29	Thermocompressor was used for fabrication and mixer prior to compression	[73]
SR	SAl_2_0_3_ + GNPs	N/A, 90 wt, 1 wt	3.37	Synergistic effect was observed	[73]
Epoxy	CF	55.9 v_f_	0.642	Vacuum bagging and hand layup process	[74]
Epoxy	CF + Cu + CNTs	56.9 v_f_, 27.16 wt	2.519	Vacuum bagging and two step method	[74]
Epoxy	CF + Cu + CNTs	56.3 v_f_, 15.7 wt	2.948	Vacuum bagging and one-step method with + charge CNTs	[74]
Epoxy	CF + Cu + CNTs	56.4 v_f_, 14.2 wt	2.732	Vacuum bagging and one-step method—charge CNTs	[74]
Bisphenol-A epoxy	CF	N/A	0.54	VARI, No fillers	[75]
Bisphenol-A epoxy	CF + GNPs	N/A, 0.5 wt	0.84	VARI, GNPs fillers and Spraying method	[75]
PA-6	BF	N/A	~0.19	No filler and twin-screw extruder	[76]
PA-6	BF + CNTs	N/A	~0.22	Filler and no coupling agent	[76]
PA-6	BF + PDA + CNTs	N/A	~0.24	Filler and PDA coupling agent	[76]

CF, carbon fibers; CNFs, carbon nanofibers; Cu, copper; CNTs, carbon nanotubes; tCNTs, CNTs treated with H_2_SO_4_; CM, compression molding; GF, graphene foam; GFs, glass fibers; MWCNTs, multi-wall carbon nanotubes; PDA, polydopamine; PDMS, polydimethylsiloxane; PEEK, poly ether ether ketone; VARI-SAl_2_0_3_, spherical alumina vacuum-assisted resin transfer infusion; N/A, not available.

**Table 5:** Electrical properties of different composites.

Type	Polymer	Fillers	Filler (%)	Electrical Properties	Additional Information	Ref.
Nanocomposite	PDMS- urea	Graphene	12 wt	81.5 S/m	Tetrahydrofuran used as solvent and Solvent casting	[80]
	CNFs	GNPs	50 wt	4057.3 S/m	Vacuum assisted filtration process	[81]
	PP/PANI	GNPs	3 phr	4.1 × 10^−1^ S/cm	Extrusion and 92% of PP and 8% of PANI	[82]
	Pullulan	TOCNS + CNTs	0.5 *w*/*v*, 5 wt	0.015 S/mm	Solution casting	[83]
Multiscale composite	PEEK	CF + Graphene	1.3 wt	0.008 S/cm	Spray method and hot press methods In plane	[84]
	PEEK	CF + Graphene	1.3 wt	0.037 S/cm	Spray method and hot press methods Through thickness	[84]
	Epoxy	CFB	40 vol	0.42 × 10^−3^ S/cm	Hot pressing and no GO	[85]
	Epoxy	CFB + PPY/GO	40 vol, 0.5 wt	6.53 × 10^−3^ S/cm	Hot pressing and coated GO with PPY	[85]
	Epoxy	CFB + PEDOT/GO	40 vol, 0.5 wt	6.2 × 10^−3^ S/cm	Hot pressing and coated GO with PEDOT	[85]
	PE	WF, CNTs	52, 12 wt	6 × 10^5^ Ω.m	High speed mixer and extruder	[86]

CF, carbon fibers; CFB, carbon fabric; CNFs, cellulose nanofibrils; GNPs, graphene nanoplatelets; GO, graphene oxide; PANI, polyaniline; PDMS, polydimethylsiloxane; PE, polyethylene; PEDOT, poly(3,4-ethylene dioxythiophene); PPE, polyether ether keytone; phr, parts per hundred resin; PP, polypropylene; PPY, poly pyrrole; RGO, reduced graphene oxide; TOCNS, TMPO cellulose nanofibrils; WF, wood fibers.

**Table 6:** Electrical and thermal conductivities of different composites.

Polymer	Fillers	Filler (%)	Electrical Properties	Thermal Conductivity (W m^−1^ K^−1^)	Additional Information	Ref.
Epon 828	-	-	4.3 × 10^−15^ S/m	-	-	[89]
Epon 828	Graphene	1.0 wt	2.6 × 10^−6^ S/m	-	Hand layup and Compression molding	[89]
Epon 828	CFB	-	5.6 × 10^−4^ S/m	0.68	Hand layup and Compression molding	[89]
Epon 828	Graphene +CFB	1.0 wt	13.1 × 10^−4^ S/m	0.72	Hand layup and Compression molding	[89]
TPU	MXene	28.6 wt	1600 S/m	6.31	In plane and through plane and layer-by-layer spraying	[87]
PP	GNPs-5	6 wt	1.56 S/m	-	Coating and compression molding	[91]
PP	GNPs-25	1.5 wt	9.20 × 10^−1^ S/m	-	Coating and compression molding	[91]
PP	GNPs-5	9.1 wt	2.02 × 10^−8^ S/m	-	Melt blending and extrusion	[91]
PP	GNPs-25	2.0, 16.7 wt	3.23 × 10^−15^ S/m	~0.85	Melt blending and extrusion	[91]
Epoxy	CF+GNPs	5 wt	0.60 S/m	~0.9	Three-way mill and VARI	[90]
-	GNF+CF prepregs	-	1799.45 S/cm	425	Autoclave woven and film without holes	[92]
-	GNF+CF prepregs	-	1364 S/cm	-	Autoclave woven and film with holes	[92]
Epoxy	CFB woven	-	2–3 Ω/sq	200	No addition of nanoparticles	[93]
Epoxy	CFB woven + (CNTs and GNPs)	-	1.03 × 10^−3^ Ω/sq	-	Addition of CNTs	[93]
Epoxy	CFB woven + (CNTs and GNPs)	-	3 × 10^−4^ Ω/sq	1500	Addition of CNTs and GNPs	[93]

CF, carbon fibers; CFB, carbon fabric; GNF, graphite nanoflakes; GNPs, graphene nanoplatelets; PP, polypropylene; RGO, reduced graphene oxide; TPU, thermoplastic polyurethane; VARI, vacuum-assisted resin infusion.

**Table 7:** Non-extensive comparison between state-of-the-art nanomaterial-based thin heaters.

Nanomaterial	R_S_ (Ω/sq)	T_op_ (%)	Temperature (°C)	Voltage (V)	Response Time (s)	Substrate	Area (cm^2^)	Method	Ref.
SWCNT	580	79	80	12	<50	PET/Glass	4 × 4	Vacuum filtration	[109]
MWCNT	349	71	58	10	N/A	PET/Glass	1.2 × 0.9	CVD	[108]
Graphene + AuCl_3_	43	89	100	12	70	PET	4 × 4	CVD	[119]
Graphene + AuCl_3_	66	90	110	12	200	Glass	2 × 2	Roll, CVD	[121]
AgNW	33	90	55	7	200	Glass/PEN	2.5 × 2.5	Spin coating, airbrush spraying	[126]
PEDOT:Sulf	57	88	100	12	383	Glass	2.5 × 2.5	Spin-coating	[131]
AgNW + SWCNT	20	90	150	15	150	PC	N/A	Spray-coating	[133]
AgNW + GO	27	80	230	15	150	Quartz	2.5 × 2.5	Vacuum filtration	[134]
AgNW + GO	26.9 *	73	84	12	N/A	Glass/PET	2.4 × 5	Spray-coating	[139]
AgNW + PEDOT:PSS	4	70	85	4	50	Glass/PET	5 × 6	Spin-coating, Doctor-blading	[135]
AgNW + PEDOT:PSS	9.4	89	120	6	20	PET	N/A	Spin-coating	[136]
AgNW + RH polymer	10	81	120	5	40	-	2.5 × 4	Solvent casting, Coating	[137]

R_S_, sheet resistance; T_op_, optical transmittance at 550 nm; PET, polyethylene terephthalate; CVD, chemical vapor deposition, PEN, poly(ethylene naphthalate); PC, polycarbonate; GO, graphene oxide; N/A, not available. * Calculated from author’s values.

**Table 8:** Non-extensive comparison between state-of-the-art nanomaterial-based heating textiles.

Material	Textile	Method	R_S_ (Ω/sq)	Temperature (°C)	Voltage (V)	Ref.
MWCNT	Cotton	Dip-dry coating	1.67 × 10^3^	70	60	[154]
SWCNT	Cotton	Dip-dry coating	439	78	20	[155]
AgNW	Cotton	Dip-dry coating	2.5	50	5	[156]
PEDOT:PSS + SDS	Cotton	Blending + dipping	24	99.6	12	[158]
Graphene + PU	Cotton	Spray-coating	N/A	162.6	12	[159]
Graphene oxide + PEDOT:PSS	Cotton	Dip-dry coating	153	41	15	[161]

R_S_, sheet resistance; PU, polyurethane; N/A, not available.

## Data Availability

Not applicable.
